# Stochastic Time Models of Syllable Structure

**DOI:** 10.1371/journal.pone.0124714

**Published:** 2015-05-21

**Authors:** Jason A. Shaw, Adamantios I. Gafos

**Affiliations:** 1 MARCS Institute, University of Western Sydney, Penrith, New South Wales, Australia; 2 School of Humanities and Communication Arts, University of Western Sydney, Penrith, New South Wales, Australia; 3 Faculty of Human Sciences, University of Potsdam, Potsdam, Germany; 4 Haskins Laboratories, New Haven, Connecticut, United States of America; Northeastern University, UNITED STATES

## Abstract

Drawing on phonology research within the generative linguistics tradition, stochastic methods, and notions from complex systems, we develop a modelling paradigm linking phonological structure, expressed in terms of syllables, to speech movement data acquired with 3D electromagnetic articulography and X-ray microbeam methods. The essential variable in the models is syllable structure. When mapped to discrete coordination topologies, syllabic organization imposes systematic patterns of variability on the temporal dynamics of speech articulation. We simulated these dynamics under different syllabic parses and evaluated simulations against experimental data from Arabic and English, two languages claimed to parse similar strings of segments into different syllabic structures. Model simulations replicated several key experimental results, including the fallibility of past phonetic heuristics for syllable structure, and exposed the range of conditions under which such heuristics remain valid. More importantly, the modelling approach consistently diagnosed syllable structure proving resilient to multiple sources of variability in experimental data including measurement variability, speaker variability, and contextual variability. Prospects for extensions of our modelling paradigm to acoustic data are also discussed.

## Introduction

The relationship between symbolic phonological structure and experimental phonetic data presents a specific case of a general challenge for modern cognitive science—the development of concepts and tools relating discrete and continuous aspects of a cognitive system. For the case of consonants and vowels, the phonological units to which most probabilistic modelling work has been devoted, general tools from statistical pattern analysis have gained traction on the problem of relating continuous phonetic dimensions to phonological categories [1–5]. Moving beyond phonemes to the syllabic level of phonological organization introduces new challenges, which we take up in this paper.

Syllables do not generally signal differences in meaning, except perhaps in cases involving presence vs. absence of a morpheme boundary, e.g., *nightrate* vs. *nitrate*, *help us nail* vs. *help a snail*. Rather, they impart organization to the units of phonological contrast. Syllable structure and the organization that syllables impart on spoken language remains stable across a range of phonemes. Thus, *plea*, *tree* and *glee* share a uniform syllabic structure—they are single syllables—independent of the physiologico-acoustic events that take place during the production of these words. Evidence for syllabification has typically come from phonological argumentation within generative linguistics [6–8]. More recently, on the experimental side, there is mounting evidence that linguistic and specifically syllabic structure shapes the continuous low-level temporal organization of articulatory movements during speech [9–14]. Hall [15] remarks that the prospect of assessing syllable structure from patterns of articulatory movement represents “an entirely new approach to studying syllables.” However, there are conflicting data and an on-going debate about the degree to which syllabic organization shapes the phonetic signal [16,17].

Drawing on phonology research within the generative tradition, complex systems theory, and stochastic methods, we develop a modelling paradigm linking discrete phonological structure, expressed in terms of syllables, to phonetic data acquired with 3D electromagnetic articulography and X-ray microbeam methods. We illustrate the paradigm with a model revealing the predictions of different syllable types and account for a number of previously observed experimental results, including both the typical phonetic properties of simplex and complex syllable parses but also the conflicting data, cases in which phonetic properties change under conditions we make precise.

Our approach combines symbolic and dynamical formal methods. Different syllabic parses of a phoneme string are mapped to distinct temporal configurations, using the concept of coordination topology, which expresses the temporal organization of phonological form [18]. Coordination topologies act as mutually exclusive independent variables in our modelling paradigm—this is the symbolic part. Using concepts from the study of complex systems, coordination topologies correspond to the essential variables describing the qualitative aspects of phonological form. The task is to identify the topology accounting for the most variability in the experimental data. The crucial dynamical component offers ways to understand the fact that the same topology, the same qualitative structure, can correspond to a range of continuous manifestations as non-essential parameters change. The fitting problem is therefore one of finding the combination of essential and non-essential variable settings that best accounts for the variability in the experimental data.

In comparison to past approaches, we take an opposing perspective on the overarching quest of relating continuous measurements to higher level units of cognitive organization. Past approaches either have asserted that there is no systematic relation between abstract phonological organization and phonetic indices ([7] pages 16–17) or have sought and sometimes failed to identify invariant patterns in speech data [19,20]. With respect to Kahn’s stance [7], an alternative approach, the one pursued in this work, is to appreciate that the relation between abstract phonological organization and phonetic indices may not be straightforward and undertake the task of developing tools enabling the systematic study of this relation. With respect to the second past approach, instead of attempting or hoping to discover invariance, our approach seeks to explain variability by positing different qualitative organizations (syllables) and mapping these to a range of quantitative manifestations as phonetic parameters change. This may appear as a retreat from the search for invariant reflexes of phonological structure in the quantitative phonetic record. But our approach is in fact stronger because it allows us to expose the conditions under which qualitative phonological form may or may not map to any given range of phonetic parameters. The key is in developing the appropriate substrate for making explicit the full range of the relation between the qualitative and the quantitative. In this approach, we can uncover the conditions under which phonetic parameters remain stable but also ask questions about how such parameters change under different conditions. This characteristic of our approach in particular enables us to diagnose syllable structure in cases of high variability in the experimental data, including cases in which past phonetic heuristics are known to break down.

The remainder of this paper is organized as follows. Section 2 provides background on syllables and their phonetic indices. Section 3 provides an overview of methods for data acquisition and quantification. Section 4 develops our modelling paradigm and reports model fits to multiple articulatory datasets drawn from English and Arabic. Section 5 summarizes results and provides a preliminary application of our tools for diagnosing the temporal patterns subserving syllable structure to acoustic data. An extension of our approach to acoustic data would enable virtually unlimited access to speaker populations for which speech movement data would be difficult or impossible to obtain. Section 6 briefly concludes.

## Background

Syllables are fundamental units of spoken language. Linguistic theories posit syllables as foundational primitives in capturing systematic cross-linguistic sound patterns [6–8,21]. Syllables are also key constructs mediating between the abstract phonological organization of language and its phonetic encoding from the perspective of phonologists concerned with the phonology-phonetics relation, phoneticians concerned with models of phonetic implementation and psycholinguists interested in lexical access and speech planning [22–24]. In seeking correlates of syllabic organization in the phonetic signal, Stetson [25] first hypothesized that syllables correspond to the “pulses” created by contractions of the intercostal muscles, which control lung volume during speech. Later studies of pulmonary air pressure during speech [26] revealed that lung pressure is kept relatively steady over the course of the production of a sentence and that the slight variations in pressure do not correspond neatly to Stetson’s “syllable pulses”. Subsequent work turned to patterns of relative timing and in this domain there is by now substantial experimental evidence for a timing-based correspondence between prosodic phonological structure, including syllables, and articulation [9–12,14,27–36]. More specifically, the hypothesis emerged that syllables correspond to characteristic patterns of coordination or relative timing between the consonants and vowels that constitute these larger units.

In a related development but on the theory side, Gafos [18,37] introduced formal background combining a theory of constraint interaction in linguistic grammars with the theory of dynamical representations on which much of the experimental work above is based. These formal tools were applied to Arabic, paving the way to experimental work on the consonant clusters in that language [38].

Arabic and specifically Moroccan Arabic is of special interest in seeking correlates of syllabic organization in the phonetic signal, because its syllable structure departs significantly from that of other well-studied languages. In their foundational work on generative phonology, Chomsky & Halle ([39] page 354) wrote that obstruent consonants (stops, fricatives and affricates) cannot form syllables by themselves or in combination with other consonants. However, subsequent theoretical work provided considerable converging evidence from phonotactics, morphology and versification that in some languages syllables are composed entirely of consonants [40–44]. Moroccan Arabic is a remarkable illustration of this case. For instance, in this language the string of consonants [nx.dm] ‘I work’ is claimed to contain two syllables and [xs.sk.tft.tʃ.fs.st.ta] ‘you have to inspect at six o’clock’ is claimed to contain seven syllables (‘.’ marks syllabic divisions). In Moroccan Arabic and other modern North African Arabic dialects [45], vowelless syllables arose from Classical Arabic through a set of vowel deletion processes that can be stated succinctly with reference to syllables: vowels in open syllables were deleted ([ki.taab] ‘book’ → [k.tab]), vowels in closed syllables were reduced to a schwa-like vocoid ([min.bar] ‘Imam’s podium’ → [m^ə^n.b^ə^r]) and long vowels were shortened ([ga.li:h] ‘he grilled’→ [g.lih])[46]. As a result of these processes, a large number of word-initial consonant clusters were created. Some of these have a rising sonority profile, as in /glih/ above or /flan/ ‘someone’, where the low sonority /g/ or /f/ are followed by the higher sonority liquid /l/. Sonority sequencing is a key concept in syllable structure [47–49]. Specifically, a rising sonority profile as in /gl/ or /fl/ is prototypical of syllable onsets cross-linguistically, and sonority sequencing is argued to underlie the processing of consonant clusters in both production and perception (for production see [50]; for perception see [51]). However, many other clusters in Moroccan Arabic do not conform to this sonority profile. Even limiting attention just to two-consonant sequences of stops at the word-initial position, all possible combinations of labial, coronal and dorsal consonants are attested, e.g., [kt], [gd], [dg], [kb], [bk], [tb], [bt]. Crucially, the syllable structure assigned to the latter clusters is the same as that for rising sonority profile clusters. That is, [d.bal] ‘to fade’ and [k.tab] ‘book’ are like [g.lih] and [f.lan] in that they are all two syllables (as before ‘.’ marks syllable divisions). This pattern of syllabification contrasts with English where strings such as /kru/ ‘crew’ or /gli/ ‘glee’ are parsed into a single syllable with a COMPLEX two-consonant cluster as its onset [6]. In Moroccan Arabic similar strings are claimed to be parsed into two syllables, e.g. /kra/ ‘rent’→ [k.ra], /skru/ → [sk.ru] ‘they got drunk’, /glih/ → [g.lih] ‘he grilled’. In these Arabic forms, the syllables with the vowels [a], [u] and [i] can only include a single consonant as their onset ([42] page 252; [45] pages 159–160), hence SIMPLEX onsets, and the syllables preceding these consist entirely of consonants [k], [sk] and [g]. A range of phonological facts provide converging evidence for this syllable structure in Moroccan Arabic. For example, patterns of seemingly puzzling variation in the phonetic forms of Moroccan Arabic words are explained parsimoniously by making reference to a ban on complex onsets. Thus, the word for ‘he sprinkled’ can be produced as [dr.dr] or [d^ə^r.d^ə^r], with a variably present voiced vocoid [^ə^], but not as [dr^ə^.dr^ə^] ([42] page 228). This variation is explained by stating that variably present voiced vocoid, [^ə^], can only occur after syllable onsets; [dr^ə^] is not possible because [dr] is not a legal syllable onset. The distribution between high vowels and glides is also cleanly captured with reference to simplex onset syllables. For example, the singular form of ‘son’ is [wld] and cannot be produced as [uld]; the plural, formed by mapping the same sequence of consonants to a CCaC form, is [u.lad] ‘sons’ and cannot be produced [wlad]. The alternation between [w] and [u] follows from a ban on complex onsets. Because [wl] cannot be an onset, [w] is parsed into a separate syllable and surfaces as [u] in accordance with the broader cross-linguistic distribution of vowel-glide pairs (specifically, the generalization that vocalic features, shared in the vowel-glide pairs such as [u]~[w] and [i]~[y] surface variantly but systematically as a vowel in syllable nucleus position and as a glide elsewhere). Finally, there has been substantial work on Moroccan Arabic versification which also supports the conclusion that this language bans complex onsets [42,52]. In Malħun songs, which conform to strict syllabic templates, word-initial consonant clusters cannot occupy a single beat. Such clusters are always split so that the first consonant, e.g., [g] of [glih], counts as an independent syllable ([42] page 252–253). In sum, Moroccan Arabic permits a rather complex set of consonant clusters and assigns a different syllabic organization to such clusters from English. This language and its contrast to English therefore provide an excellent empirical domain for the development of methods to diagnose phonological and specifically syllabic organization in the phonetic signal.

**Fig 1:**
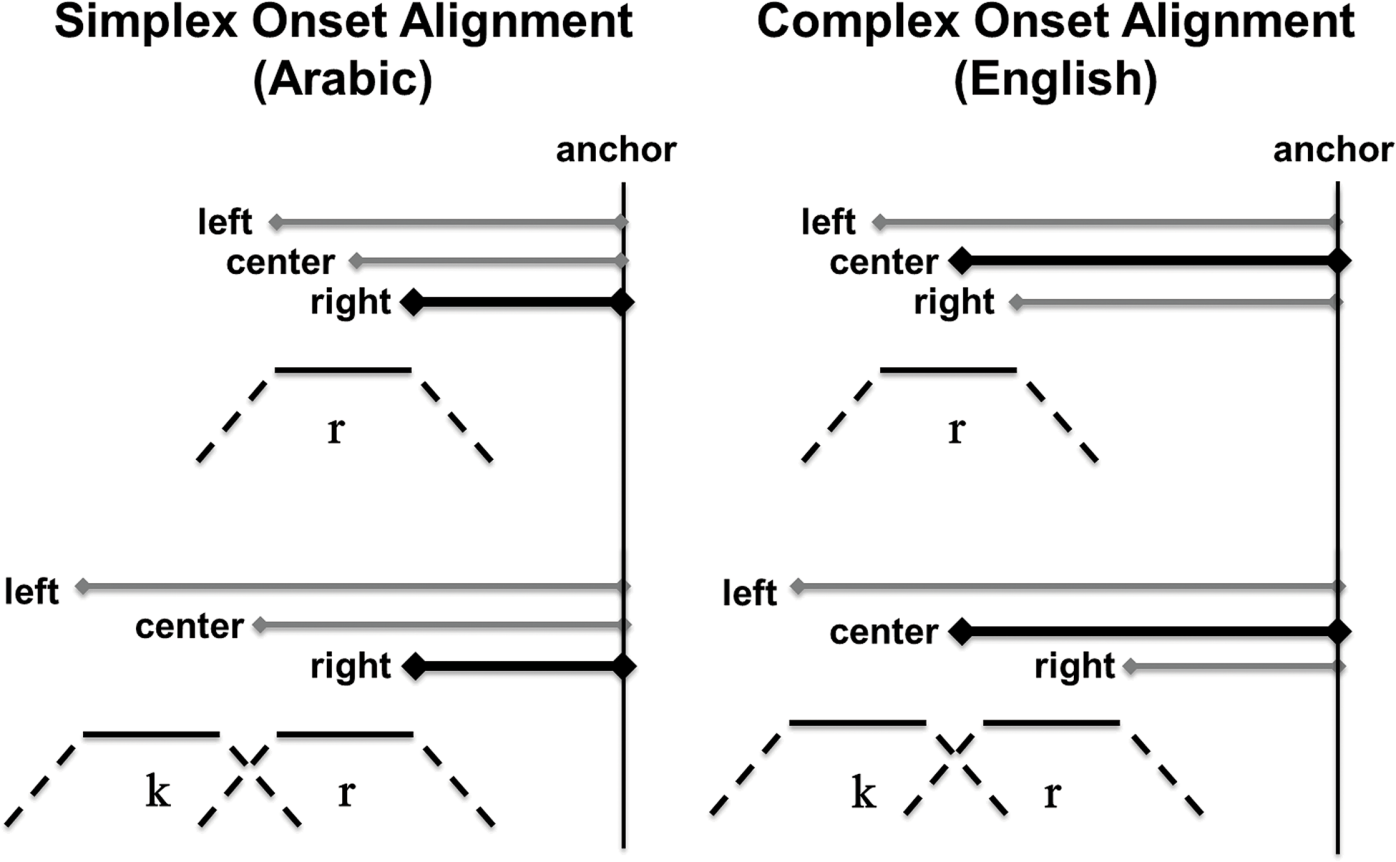
Temporal alignment schemas. Schematic representation of three intervals, left edge to anchor (LE-A), center to anchor (CC-A) and right edge to anchor (RE-A), delineated by points in an initial single consonant, /r/ (top row), or consonant cluster, /kr/ (bottom row), and a common anchor (A). The alignment schema on the left shows simplex onset organization. The schema on the right shows complex onset organization. They key difference between simplex and complex alignment is in the patterns of change in the intervals across /r-/ (top) and /kr-/ (bottom) initial words. On the left (simplex), looking across the top and bottom schemes, RE-A remains stable while LE-A and CC-A increase. On the right (complex), looking across the top and bottom schemes, CC-A remains stable while LE-A increases and RE-A decreases.

Contrasts in syllable structure such as that between Arabic and English illustrated above are known by now to have consequences for temporal organization. Differences in temporal patterning between Arabic and English emerging from past work [9,13,31,53] are schematized in Fig 1. The temporal alignment schemas shown in this figure illustrate distinct temporal organizations for sequences of consonants. The left side of Fig 1 shows simplex onset alignment, the temporal alignment pattern observed for Arabic. The right side of the figure shows complex onset alignment, the pattern observed for English. Note that both schemas exhibit the same timing in CV syllables (Fig 1, top), but differ in how the CCV sequence (Fig 1, bottom) is timed relative to the CV sequences. The temporal differences illustrated schematically in Fig 1 can be captured quantitatively through analysis of how temporal intervals change across CV and CCV syllables. Arabic, a language in which consonant sequences cannot begin the same syllable, parses CCV as [C.CV] and exhibits the simplex onset alignment pattern (left). English parses CCV as [CCV] and exhibits the complex onset alignment pattern (right). The depicted patterns are canonical temporal schemes to which exceptions have been found. Both the canonical schemes as well as exceptions to them are of central concern in this paper.

In Fig 1, the temporal life of individual segments, ***r***, ***k***, is represented by three lines: a dotted line corresponding to movement toward constriction, a solid line corresponding to constriction duration and another dotted line corresponding to movement away from constriction. For each alignment schema, two words differing in the number of initial consonants, ***r***, ***kr***, are shown. In addition, the figure shows three intervals for each word. The intervals are left-delimited by the left edge, right edge and center of the single consonant or consonant cluster and right-delimited by a common anchor (a) on the following vowel (exact definitions of these ‘landmarks’ are given in the next section). The difference between simplex and complex onset alignment can be discerned by observing how the duration of these intervals changes across words, e.g. *rue*, *crew*. Simplex onset alignment corresponds to a pattern whereby the right edge to anchor (RE-A) interval is more stable than the center to anchor (CC-A) and left edge to anchor (LE-A) intervals [10,13]. In Fig 1, left, the relative stability of the RE-A interval is indicated by the constant length of the horizontal line drawn between the right edge and the anchor. In reality, as stability is assessed across word types and multiple repetitions of each word, the RE-A interval is not constant. It is patterns of relative stability among intervals that are schematized here and such patterns can be statistically assessed as done in the references cited above. For complex onset alignment, a different pattern is found whereby the CC-A interval is more stable across words than the LE-A and RE-A intervals [9,10,31]. As shown in Fig 1, right, in complex onset alignment it is the horizontal line between the center of the cluster, or “c-center”, and the anchor that remains constant across the two words. These results concur with independent arguments from phonological theory that Arabic disallows complex consonant clusters as syllable onsets whereas English permits them [7,42,54]. Accordingly, in Arabic the string /kra/ ‘rent’ would not be just a single syllable. Rather, [k] would be in a different syllable from [ra]. Intuitively, we can describe the correspondence between these theoretical ideas and the data patterns of Fig 1 as follows. Since in Arabic, it is only the immediately prevocalic consonant that is in the same syllable as the vowel, their timing relation should remain unperturbed when another consonant is added to the beginning of the word. Thus, no change in the interval between the prevocalic consonant and the vowel is expected (Fig 1, left). In English, in contrast, since the added consonant is incorporated into the same syllable as the rest of the segments, the timing relation between these segments must change to accommodate the extra member of the syllable. Thus, we expect the interval between the prevocalic consonant and the vowel to change when another consonant is added (Fig 1, right). These previous studies and other related ones in the research above provide methods for exploring the syllabic structuring of phonological form in terms of temporal patterns in the phonetic signal.

In our work, these results provide the starting point for a systematic experimental, modelling and analytical approach for studying the relation between the mental organization of language in terms of syllables and its phonetic reflexes. A key tenet of this integrative approach is a commitment to phonological theory. Symbolic syllable structure remains constant across different physical instantiations of syllables. In English, [gli] ‘glea’ and [spa] ‘spa’ share a uniform syllable structure—they are both single syllables—despite the different articulators involved in their production. Likewise, in Morrocan Arabic, [u.lad] ‘sons’ and [g.lih] ‘he grilled’ pattern together in the phonology—as sequences of two syllables—despite the different articulators involved in production. Uniformity in syllable structure is independent of the physiologico-acoustic events that take place during the production of these words. Our approach to modelling the temporal basis of syllables is to posit different fixed temporal organizations or coordination topologies (following [18,55]) that correspond to different hypothesized syllable structures. In modelling, we capture variability introduced by articulator differences, and numerous other sources, through the incorporation of non-essential model parameters. Varying these parameters reveals the range of continuous indices predicted by the essential variables, in our case, syllable structure. This allows us to derive canonical patterns in experimental data, such as those depicted in Fig 1, but also patterns which deviate systematically from the expected temporal manifestations. Before developing our modelling paradigm in greater detail, we first describe the experimental data.

## Experimental Data

### Data acquisition

The experimental data were acquired using electromagnetic and X-ray microbeam technologies, which allow the tracking of fleshpoints on speech articulators with high spatial-temporal resolution. In the electromagnetic articulography method (henceforth, EMA), an electromagnetic field is used to track movements of small receiver coils glued on the speech articulators, i.e. lips, tongue tip, tongue dorsum and jaw [56]. Transmitter coils, three coils (in two-dimensional EMA) or six coils (in three-dimensional EMA) [57], produce alternating magnetic fields at different frequencies in the range of about 10 kHz. The fields from the transmitter coils pass through the receiver coils and generate an electric signal. The voltage of this signal is related to the distance and orientation of the receiver relative to the transmitter coils. This relationship is used to calculate the position of the receivers as a function of time, permitting access to the fine details of the spatial and temporal properties of vocal-tract action during speech. The voltages in the receiver coils are captured at a sampling rate of 200 Hz. Audio data are also collected in parallel with the articulatory data. In the X-ray microbeam method, a flying spot X-ray microbeam generator emits a narrow beam of high energy X-rays [58]. High-speed computer control of this beam tracks gold pellets glued on the speech articulators [59–61]. The X-ray microbeam method pre-dates EMA, but produces fully comparable datasets [62]. The datasets modelled in the paper include already published results on Arabic [13,63] and English [9], publically available data [61] and new data. The procedures for new data collection were approved by the Ethics Committee at the University of Potsdam, and written consent was obtained from all participants.

### Quantifying temporal stability

In order to quantify temporal organization, we decompose articulatory movements corresponding to consonants and vowels into a series of landmarks ([18] page 276). These include Start: the onset of movement toward an articulatory target; Target: achievement of an articulatory target; Release: the onset of movement away from an articulatory target; and End: the offset of controlled movement away from an articulatory target. For each consonant or vowel, these landmarks are identified by automatic algorithm with reference to the velocity signal of the relevant articulator. The receiver or pellet used to delineate the movement associated with a consonant is the one corresponding to the consonant’s primary oral articulator, e.g. the tongue tip for [d], the lower lip for [f], and the tongue back for [g]. The algorithm locates the timestamp at which the instantaneous velocity exceeds, in the case of start and Release, or falls below, in the case of Target and End, a set percentage of the velocity peak associated with movement toward or away from an articulatory target [64].

**Fig 2:**
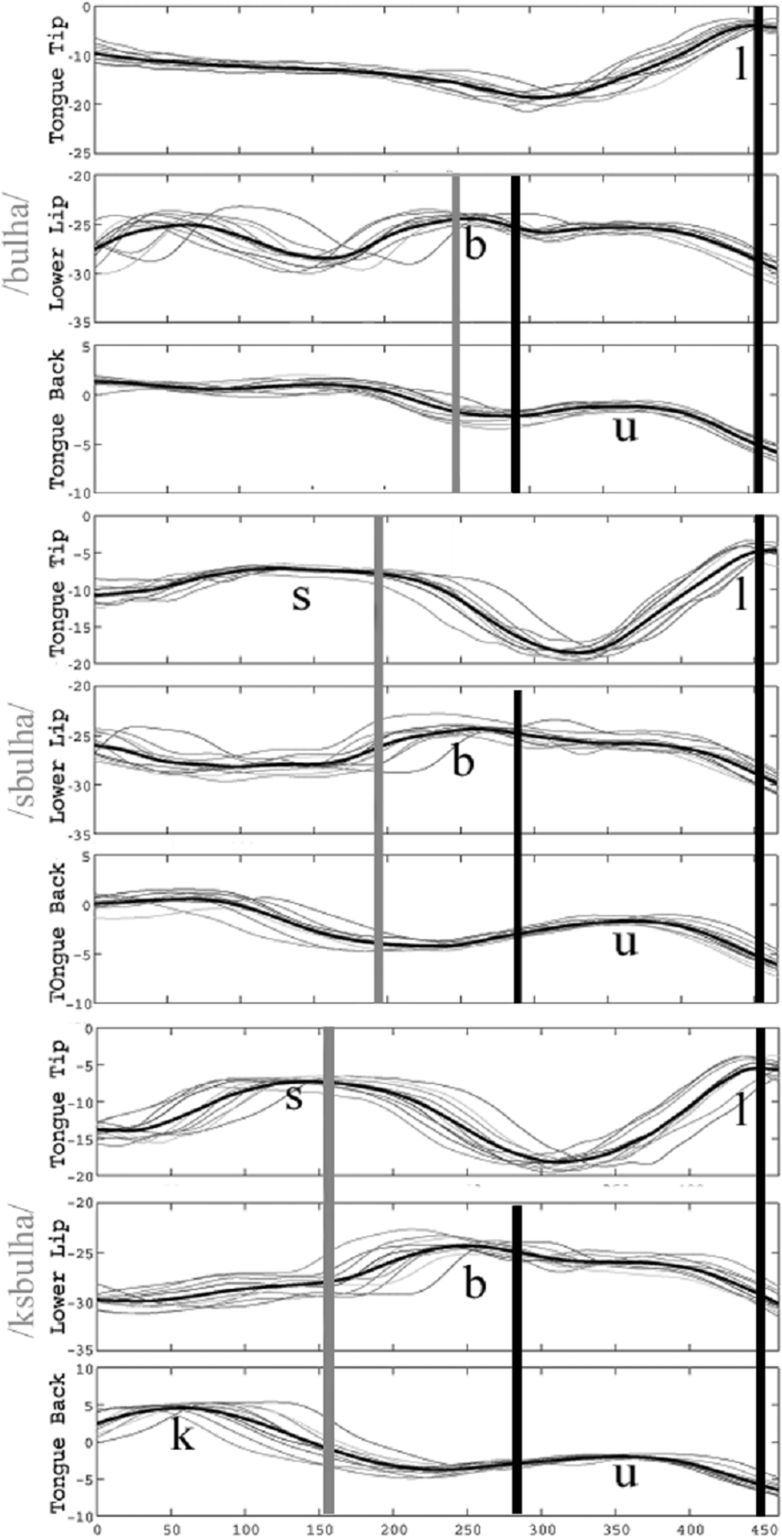
Illustration of temporal alignment in Arabic. Positional signals in the y-dimension for 3 different receivers, tongue tip, lower lip and tongue back, for 10 repetitions each of *bulha*, *sbulha*, *ksbulha*. The leftmost vertical line (grey) demarcates the center of the initial consonant cluster (or single consonant as in *bulha*). The middle vertical line demarcates the release of the prevocalic consonant [b]. The rightmost vertical line demarcates the point of inferred maximum constriction in the post-vocalic consonant [l]. Reprinted from [13] under a CC BY license, with permission from Cambridge University Press (S2 File), original copyright 2011.

Extracting landmarks from speech movements using this procedure yields a series of timestamps. These timestamps are used to quantify patterns of temporal organization corresponding to distinct syllable parses. Fig 2 provides an illustration of how temporal landmarks parsed from the velocity signal are used to define structurally relevant temporal intervals (as schematized in Fig 1) for three Arabic words *bulha*, *sbulha*, and *ksbulha* (‘her urine’, ‘her ear of grain’, ‘they owned it for her’). For each word, the positional signal in the *y*-dimension (up-down movement) is shown. Only the *y*-dimension is shown here for simplicity in presentation. Actual data analysis is based on both the vertical and horizontal (front-back) movements of articulators. Each panel of Fig 2 shows ten trajectories (grey lines) corresponding to ten repetitions of the word along with a highlighted ensemble average (black line). Three vertical lines are drawn for each word. The rightmost line corresponds to the anchor, the timestamp that right-delimits the temporal intervals of interest. In this example, the anchor is the maximal vertical displacement of the tongue tip movement corresponding to the segment [l]. That segment was chosen because it is at the end of the hypothesised syllabic unit and is shared across all stimuli (it appears after the vowel in each stimulus word). We refer to this point as C^Max^. In the analyses to follow, we use the articulatory landmarks of either C^Max^ or V^End^, the offset of the vowel, as anchor points. Changing the anchor point from C^Max^ to V^End^ increases the amount of variability in the intervals. Later on, we show that our models can capture how such increases in variability influence phonetic heuristics for syllables.All thirty data tokens in Fig 2 (three words, ten repetitions) are aligned at the C^Max^ anchor timestamp. A second vertical black line is drawn at the mean value of the Release timestamps of the lower lip constriction for the [b], *b* ^Release^ in *bulha*, *sbulha* and *ksbulha*. Another vertical line is drawn at the center of the word-initial consonant cluster. The center of a cluster is the mean of the midpoints of each consonant in the cluster, where consonant midpoint refers to the point equidistant to the Target and Release landmarks of the consonant. As Fig 2 shows, the interval between *b* ^Release^ and the anchor point does not seem to change much across *bulha*, *sbulha*, *ksbulha*. In contrast to what is observed for *b* ^Release^, the center of the consonant cluster gets farther away from the anchor point with each consonant added. This is indicated by the progressive leftward shift of the vertical grey lines from *bulha* to *sbulha* to *ksbulha*. In these Arabic datasets, then, as consonants are added at the beginning of a word, the local timing relation between the [b] and its adjacent vowel does not change much. This was schematically shown in the left panel of Fig 1. This Arabic temporal organization contrasts with the English one schematized in the right panel of Fig 1. In English, as consonants are added at the beginning of the word, the local timing between the prevocalic consonant and the vowel has been reported to change. In the right panel of Fig 1, this was shown by the progressive rightward shift of the prevocalic consonant.

The patterns illustrated in Fig 2, like those to be quantified in our data, involve comparisons of interval stability, e.g. “the interval between *b* ^Release^ and the anchor point does not seem to change much” and so on. Quantitatively, interval stability is assessed using the standard deviation (SD) of an interval’s duration and its relative standard deviation (RSD), defined as the ratio of the standard deviation to the mean. RSD is an appropriate stability index for our purposes because the mean of a timed interval is correlated with its variance [55,65], and we intend to compare the stability of temporal intervals of inherently different durations. In the remainder of this paper, we develop models to capture patterns of stability, expressed in RSD and quantified over the LE-A, CC-A, and RE-A intervals, as shown in Fig 1.

## Models

### Overview

**Fig 3:**
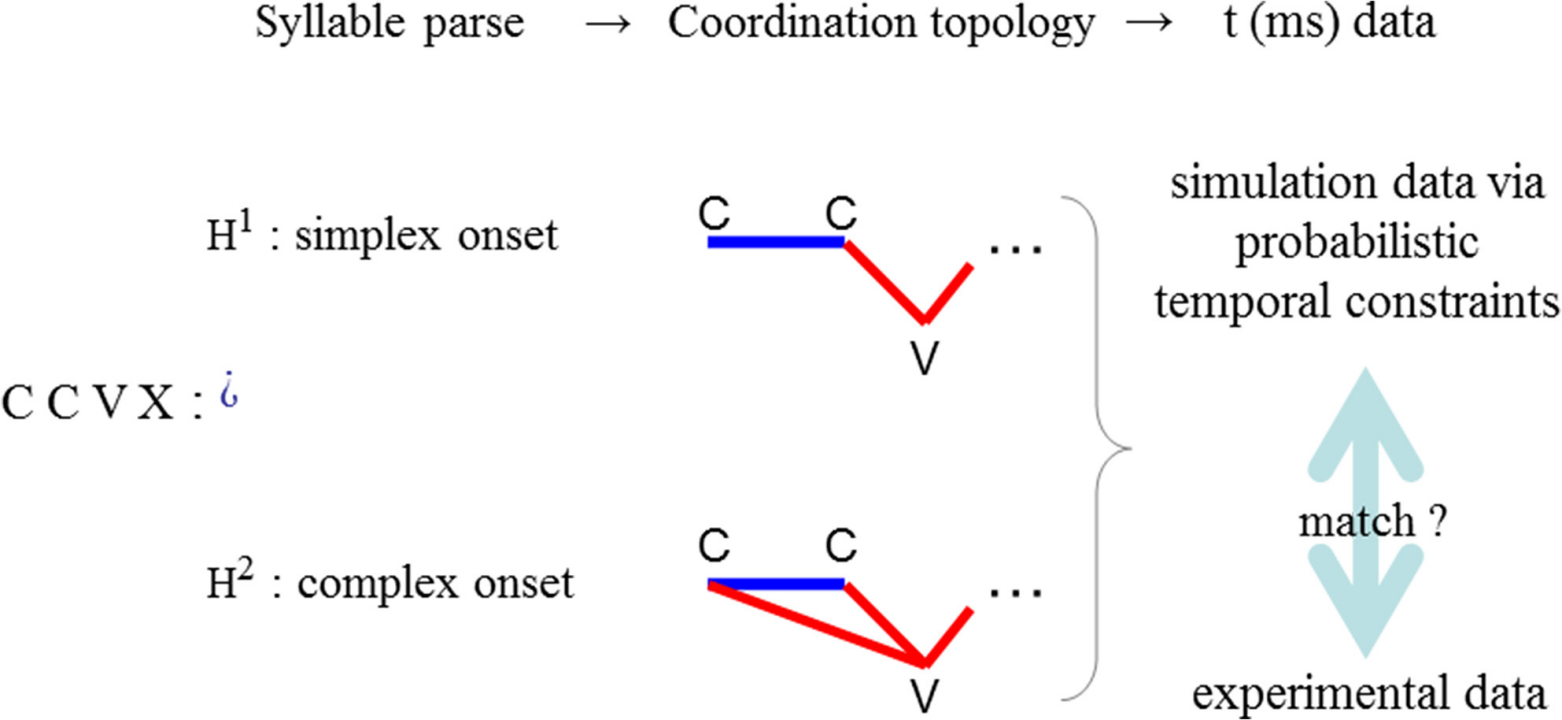
Model overview. Given any sequence of consonants and vowels, here “C C V X”, we exemplify our modelling paradigm by asking: is the sequence parsed in terms of syllables of the simplex or the complex onset type? To evaluate the two hypotheses, H^1^ vs. H^2^, the model projects coordination topologies from hypothesized syllable parses. The topology on the top/bottom embodies temporal relations of the simplex/complex onset parse. Absolute time (ms) predictions can be derived from these topologies, and their match to experimental data can be rigorously evaluated.

A schematic of the modelling paradigm is shown in Fig 3. Each syllabic parse can be mapped to a coordination topology ([18] page 316), reflecting the temporal relations underlying the segmental sequence. Two contrasting coordination topologies corresponding to a simplex onset parse (H^1^) and a complex onset parse (H^2^) of a segmental substring CCVX are shown in Fig 3. Mnemonics are ‘C’ for any consonant, ‘V’ for any vowel, and ‘X’ for any string over the C,V alphabet. These topologies specify timing relations between consonants and vowels, indicated by lines between the segments so related. Different topologies act as mutually exclusive independent variables, e.g. in the example of Fig 3, for any given CCV sequence, the parse in which both consonants are part of the onset, as per the English syllable structure, is pitted against the parse in which only the prevocalic C is included in a syllable with the V, as per the Arabic syllable structure. The task is to identify the topology accounting for the most variability in the data. For example, it is expected that for a CCV string in a language that does not admit complex onsets, the simplex onset topology would explain more variability than the complex onset topology.

From a coordination topology, our models generate temporal structure that reflects this topology. Given a set of word types, e.g. CVX, CCVX, CCCVX, our models generate articulatory landmarks defining the *plateau* of each consonant in relation to its adjacent consonants and to the vowel. The plateau of a consonant is defined as the interval demarcated by two landmarks, Target and Release. The Target corresponds to the timestamp of the achievement of the consonant’s constriction (see also Section 3.2), e.g. the timepoint where the tongue makes contact with the alveolar ridge during the formation of a [t]. Release corresponds to the timestamp of the beginning of the movement away from that constriction (section 3.2). These landmarks are generated from stochastic versions of timing relations between consonants and vowels.

Syllable structure enters crucially in the statement of these relations. In a CCV sequence, the hypothesis that it is syllabified as C.CV, with a simplex onset, dictates that the vowel Start is timed to only the immediately prevocalic consonant. The hypothesis that it is syllabified as CCV, with a complex onset, dictates that the vowel Start is timed to the center of the entire prevocalic consonantal cluster. These outcomes can be derived from interaction of competing coordination relations ([18] page 316–322). Syllabic structure then determines the timestamp of the Start landmark for the vowel. From this timestamp, we derive the timestamp of the anchor, which is found all the way at the other end of the syllable, i.e., as in the V^End^ landmark (see Fig 1 for schema), by adding a term corresponding to the vowel’s duration equal to the mean vowel duration in the experimental data. Based on this new timestamp, a set of anchor distributions is generated with the same mean but differing standard deviations. For example, some of our simulations reported below use a population of anchors in which the standard deviation of the anchor increases from 0 ms in anchor 1 to 95 ms in anchor 20 in steps of 5 ms. Anchor variability is used as a stand-in for any source of variability in the intervals spanning within and across the hypothesized syllabic constituents. Such sources include speech rate, lexical statistics, measurement error, and, of course, the segmental identity of the consonants involved. These and other yet unknown factors introduce noise in our experimental data. For instance, speech rate may vary from one stimulus production to another and lexical frequency and phonological neighbourhood density may affect variability in articulation [66,67]. Contextual predictability and repetition are also known to influence duration [68,69], and they do so independent of frequency [70]. In our paradigm, such variability is injected in the simulated data by systematically changing the standard deviation of the anchor distribution. Since it is the pattern of relative stability that is diagnostic of syllable structure, it is important that our variability manipulation affects all relevant intervals equally. Injecting variability at the anchor point ensures this because all intervals quantified in our data analyses are right-delimited by a shared anchor (see Fig 1), e.g. left edge to anchor (LE-A), center to anchor (CC-A) and right edge to anchor (RE-A).

Our description of coordination relations above is based on alignment of gestural landmarks. An alternative is coordination relations expressed in terms of phases. In models of speech production using phases [71], gestures are defined using the dynamics of second-order mass-spring systems. A gesture is associated with an abstract 360^0^ cycle. A phase corresponds to a point on the cycle of the oscillating body, and is expressed by number of degrees on the cycle. Coordination relations are expressed in terms of synchronizing phase angles. A mapping can be established between temporal organization as generated in our scheme and the phase-based description. Specifically, the spatio-temporal landmarks in the coordination relations of our model correspond to phase angles, as in Start is at phase 0^0^, Target at 240^0^, Release at 290^0^ and so on. In the phase-based description, the duration of a gesture's cycle is determined by the stiffness parameter, with lower stiffness implying lower frequency and thus longer period (duration of one cycle). Stiffness maps to plateau duration in our model. Therefore, our choice of stating coordination relations does not prevent us from relating our models to those in a phase-based description. At the same time, our choice does not force us to make additional assumptions about the relation between data and model parameters.

Specifically, instead of using phases, we employ the landmark-based scheme of stating coordination relations, because it requires fewer additional assumptions in going from data to model parameters. One can easily estimate the phonetic parameters needed to model coordination relations based on gestural landmark alignment. For a given corpus, plateau duration, inter-plateau interval, and vowel duration can be readily estimated. Estimating the parameters needed for a phase-based model, namely, stiffness and phasing relations, is not trivial or requires additional assumptions. For undamped systems, there is a direct, analytical mapping from the time domain (the coordinate system defined by the position of an oscillating body and time) to the phase plane (the coordinate system of position by velocity). Gestures, however, are assumed to be critically damped [71]. A system whose behavior is described by such dynamics does not oscillate and reaches its target after an infinite amount of time. Therefore, assumptions have to be made to import the notion of phase in stating coordination relations under critical damping. This is because the analytical relation from the time domain to the phase plane is lost, which means that calculating phase angles from actual data is not possible. On experimental results concerning inter-gestural phasing, see [20]. For applications to syllable structure and modelling results using a phase-based scheme see [32] and [72].

Finally, for phase-based schemes, coordination relations are usually expressed with two universally assumed phasing values, in-phase and anti-phase. To quantitatively fit experimental data, our models require more specific information than two phases. One reason for this is that different consonant clusters (for which their constituent consonants are sequential and thus timed anti-phase) exhibit different degrees of overlap. Thus, saying they are anti-phase is not sufficient. For our quantitative aims, we need estimates of phonetic details which are under-determined by the two phasing relations above.

To sum up the central idea, the task of evaluating syllable parses with experimental data has been formulated here as the task of fitting abstract coordination topologies to the experimental data (see Fig 3). This fitting can be expressed using two types of parameters, coordination topologies and anchor variability. In the study of biological coordination and complex systems more generally, these two parameters correspond respectively to the so-called essential and non-essential parameters describing the behavior of complex systems ([73] page 13). Essential parameters specify the qualitative form of the system under study. For us, this corresponds to the syllabic parse of the phonological string. The fundamental hypothesis entailed in positing an abstract phonological organization isomorphic to a syllable parse is that syllables are macroscopic units at the qualitative level of description. This means that syllables and the organization they impart on spoken language remain stable across the variable phonic identities of the sounds that take part in the hypothesized syllabic structuring of speech [74]. Syllable structure is independent of speech rate, the frequency of the specific words or the combinatorial probability of the particular phonic sequences that make up these words in the mental lexicon. All of these latter factors have left imprints on the articulatory patterns that are registered in experimental data. Crucially, we do not know and it may not be possible to predict for any given stimulus how each such factor or combination of factors affects the intervals to be quantified. Therefore, in formulating the modelling problem of diagnosing syllable structure in experimental data, we let variability be one of the parameters manipulated in the fitting process.

### Defining the models

The modelling paradigm described above serves to provide explicit links between qualitative phonological organization and its manifestation in terms of continuous indices such as phonetic duration and its variability. Via simulation, our models generate simulated quantitative data from hypothesized qualitative phonological structures. Then, the simulation-generated data can be compared or fitted to the experimental data.

**Fig 4:**
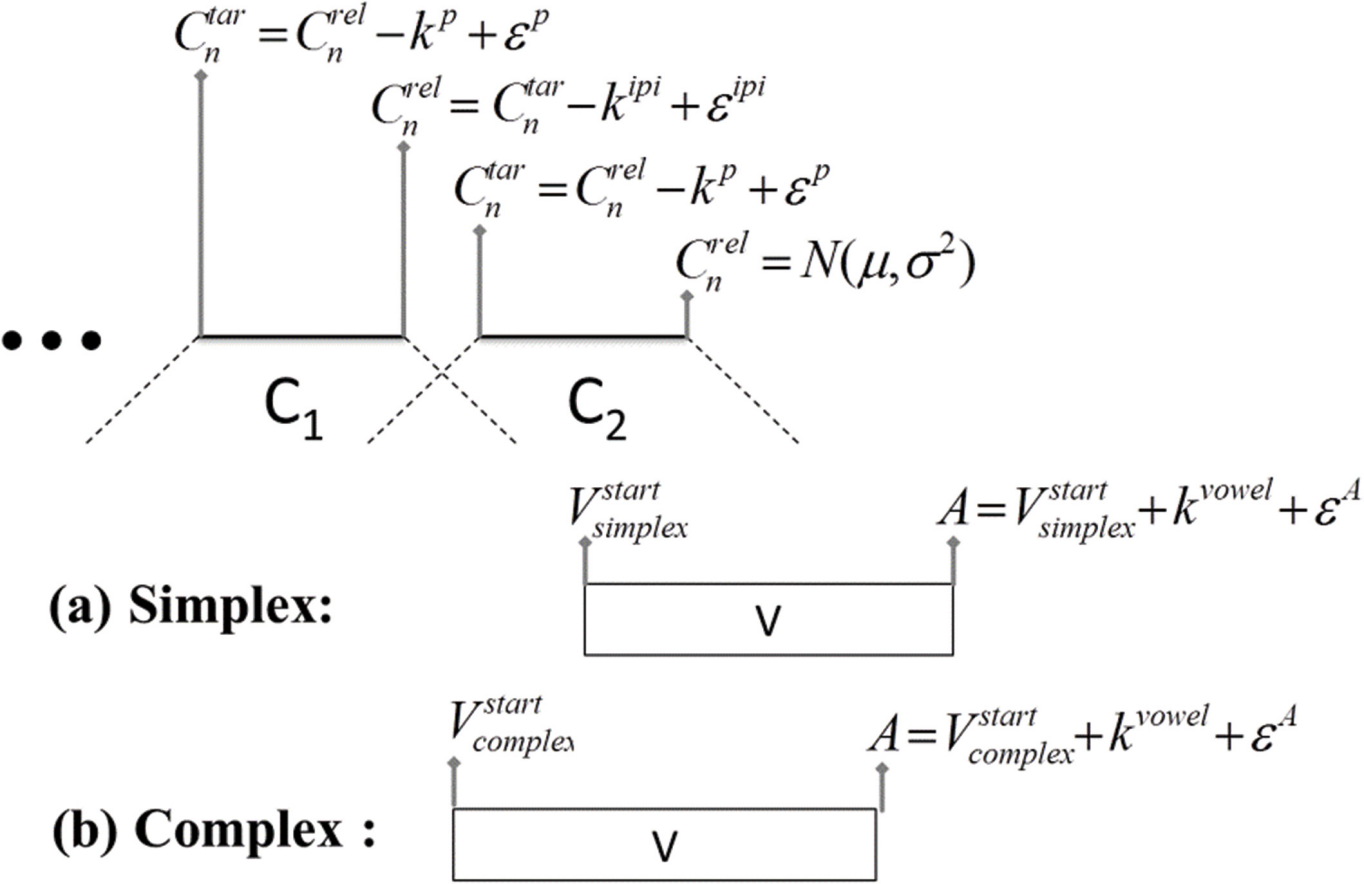
Summary of word simulation algorithm. Consonant landmarks are generated from the release of the immediately prevocalic consonant. The alignment of the vowel is determined by the syllable parse (simplex or complex). All landmarks are associated with a noise term, ɛ.

The simulation algorithm is summarized in Fig 4. Word simulation proceeds from the release landmark, $C_{n}^{r\;el}$, of the immediately prevocalic consonant, *C*_*n*_. The timestamp of the achievement of target of this consonant, $C_{n}^{tar}$, is determined by subtracting consonant plateau duration, *k* ^*p*^, from $C_{n}^{tar}$ and adding an error term specific to consonant plateau duration, *ɛ* ^*p*^. The error term is a random variable with a mean of 0 and standard deviation drawn from measurements of plateau duration in the data. Taken together, the plateau duration constant, *k* ^*p*^, and the error term, *ɛ* ^*p*^, define the distribution of plateau values in the data being modelled. Additional prevocalic consonants, e.g. C_1_ in #C_1_C_2_V, are determined with reference to the immediately following consonant. For example, the timestamp of the release of, *C*_*n*−1_, $C_{n-1}^{rel}$, is determined by subtracting the inter-plateau interval, *k* ^*ipi*^, from $C_{n}^{tar}$ and adding an error term specific to inter-plateau duration, *ɛ* ^*ipi*^. As with plateau duration, the constant for inter-plateau interval and the inter-plateau interval error term describe a normal distribution of inter-plateau intervals in the data being modelled. In this way, consonantal landmarks are simulated identically for both simplex and complex onset models. The difference between the models, as noted above, is in the alignment of the vowel relative to prevocalic consonant clusters. This alignment is dictated by syllable parse. For simplex onset organization, the start of the vowel, *V* ^*start*^, is left-aligned to the midpoint of the immediately pre-vocalic consonant. For complex onset organization, the *V* ^*start*^ landmark is left-aligned to the c-center, a point determined by the mean of the midpoints of all prevocalic consonants. This difference in vowel alignment accounts for the distinct patterns of variability characteristic of simplex vs. complex onset syllables. The anchor point, *A*, occurs at the end of the syllable and right-delimits the three intervals of interest (Fig 1: left edge to anchor, center anchor, right edge to anchor). It is simulated by adding a constant corresponding to vowel duration, *k* ^*vowel*^, to the *V* ^*start*^ landmark and adding an error term, *ɛ* ^*A*^. This final error term is varied in our simulation creating the different anchor distributions described above so that we can observe syllable-referential patterns of temporal stability at different levels of variability in the intervals.

Crucially, the only difference between the models is in terms of syllable structure. From each model, simulated data was generated for word replicas with shapes corresponding to word shapes in the corpus. In the simulated data, the relative standard deviation (RSD) of the same three intervals measured in the experimental data, LE-A, CC-A, and RE-A, was calculated for each value of *ɛ* ^*A*^. RSD was calculated by dividing the standard deviation of the interval by the mean interval duration. The actual temporal structure corresponding to a coordination topology is determined by the totality of deterministic timing relations and interactions between these, but it is also affected by non-deterministic or stochastic forces in the timing relations, as described above. Hence, the models are stochastic time models of syllables for which the statistics of the temporal organization corresponding to a syllable parse can be determined by sampling across many repetitions of actuating or simulating that parse. The RSDs so produced are then evaluated against RSDs calculated from the experimental data. In the remainder of this section, we report model hit rates for experimental data drawn from English and Arabic, languages hypothesized to differ in syllabic structure.

### Fitting the data

**Fig 5:**
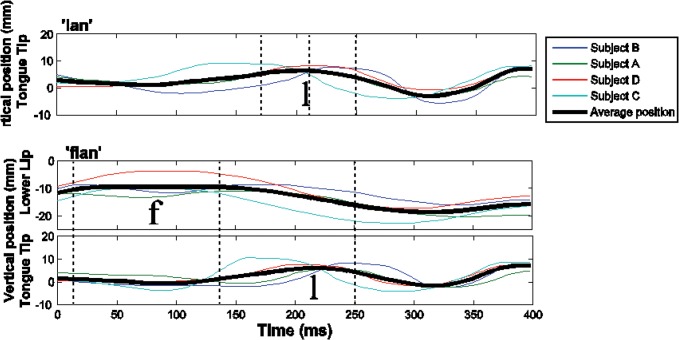
Articulatory recordings of Moroccan Arabic. The top panel shows the movement of the tongue tip during the production of *lan* ‘to become soft’ by four speakers. The bottom two panels show the tongue tip and lower lip movement during the production of *flan* ‘someone’ by the same four speakers. The colors of the lines indicate the different speakers. The thick black line shows the average trajectory across speakers. Dotted vertical lines indicate the landmarks that left-delimit the temporal intervals of interest: left edge, center, and right edge. The movement trajectory of the tongue tip is relatively consistent across *lan* and *flan* tokens. In particular, the right edge landmark is stable while the center and left edge landmarks shift to the left.

Recent work on Moroccan Arabic has reported relevant measurements of EMA data for a number of different word sets, either matched dyads, such as *tab*~*ktab*, or where possible, matched triads, such as *bulha~sbulha~ksbulha*, discussed above [13,63]. Fig 5 shows representative EMA data from Arabic. The upper panel shows the movement of the tongue tip sensor during production of the word *lan* ‘to become soft’ by four different speakers of Moroccan Arabic. The position of the tongue tip rises to form the constriction for /l/, then falls, and, finally, rises again to form the constriction for /n/. All speakers show this pattern, even though there is variation across speakers in the interval between the two vertical maxima, or consonantal plateaus. The thick black line shows the average trajectory across speakers. The bottom two panels of Fig 5 show movement of the tongue tip sensor and the lower lip sensor during production of the word *flan* ‘someone’. The individual grey lines correspond to the same Arabic speakers in the upper panel. The movement trajectories for *lan* (upper panel) and *flan* (lower two panels) are both right-aligned to the anchor. The duration of the movement of the tongue tip is relatively constant across *lan* and *flan* at both the level of individual speakers and the average across speakers. This pattern reflects simplex onset alignment, the schema laid out in the left side of Fig 1.

**Fig 6:**
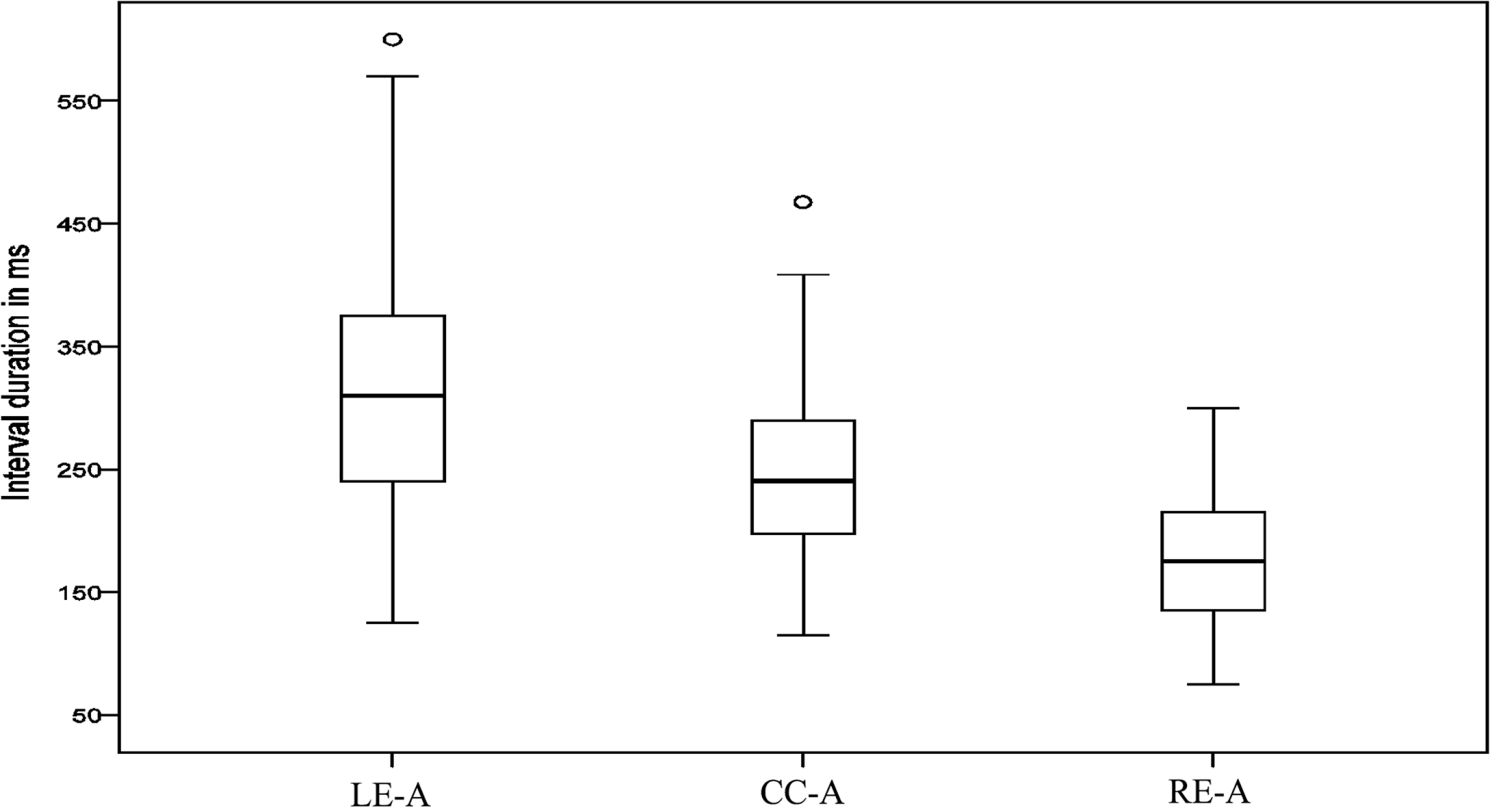
Duration of measured intervals in Arabic. Each box corresponds to 567 data points (collapsing over data reported in [13,63]). Left box: LE-A (left edge to anchor interval), middle box: CC-A (center to anchor interval), right box: RE-A (right edge to anchor interval). Intervals shown here were right-delimited by the C^Max^ anchor.

We now turn to model fitting for our first Moroccan Arabic corpus, which consists of 22 words: *bal* ‘to urinate’, *dbal* ‘to fade’, *tab* ‘to repent’, *ktab* ‘book’, *lih* ‘for him’, *glih* ‘to grill’, *bati* ‘to spend the night’, *sbati* ‘belt’, *bula* ‘urine’, *sbula*, ‘thorn’, *bulha* ‘her urine’, *sbulha* ‘her ear (of grain)’, *ksbulha* ‘they owned it for her’, *dulha* nonce *kdulha* nonce, *bkdulha* nonce, *kulha* ‘eat for her’, *skulha* nonce, *mskulha* ‘to hold for her’, *lan* ‘to become soft’, *flan* ‘someone’, *kflan* nonce. Fig 6 summarizes interval measures for this corpus. It shows the mean duration of LE-A, CC-A, and RE-A intervals for 567 data points drawn across the entire corpus. The main observation is that the variability of the RE-A interval is lower than the CC-A interval and the LE-A interval. For a complete description of the data including statistical analyses see [13,63].

In fitting the data, we assess syllable structure through patterns of interval RSDs. RSD values for the LE-A, CC-A, and RE-A intervals are simulated and compared to values in the data. The phonetic parameters in the model, plateau duration (*k* ^*p*^, *ɛ* ^*p*^), inter-plateau interval (*k* ^*ipi*^, *ɛ* ^*ipi*^), and vowel duration (*k* ^*v*^), were set to means in the data computed across word set (usually matched dyads and triads), speakers, and trials. For a given word set, we conducted 1000 simulations. On each simulation run, RSDs were generated according to a hypothesized syllable parse across different levels of anchor variability (*ɛ* ^*A*^). At each level of anchor variability (on each run) we evaluated the goodness of fit between data RSDs (three values for dyad/triad) and model RSDs (three corresponding values for dyad/triad). A *hit* was recorded for a simulation run if the goodness of fit to the data exceeded threshold at any level of anchor variability.

The procedure for determining *hits* is as follows. Using the least squares method, a line was fit to coordinates determined by pairings of experimental and simulated RSDs ($RSD_{sim}^{LE-A}$, $RSD_{data}^{LE-A}$; $RSD_{sim}^{CC-A}$, $RSD_{data}^{CC-A}$; $RSD_{sim}^{RE-A}$, $RSD_{data}^{RE-A}$). Residual sum of squares, *SS*_*residual*_, were calculated as the sum of the square distances between the RSD coordinates and the best fitting line, according to the equation *SS*_*residual*_ = ∑(*x*_*data*_ − *x*_*linearfit*_)^2^, where *x*_*data*_ is the RSD value from the data and *x*_*linearfit*_ is the closest point on the best-fitting line. Total sum of squares were also calculated using the standard equation, *SS*_*total*_ = ∑(*x*_*data*_ − *μ*(*x*))^2^, where μ(x) is the mean of RSD values in the experimental data. The sum of squares of the model, *SS*_*model*_, was obtained by subtracting the residual sum of squares from the total sum of squares: *SS*_*model*_ = *SS*_*total*_ − *SS*_*residual*_. This indicates the improvement of the linear fit computed from the simulated RSDs over the mean as an estimate of data points. An F statistic, $F=\frac{SS_{model}}{\left(SS_{residual}/df\right)}$, was then calculated by taking the ratio between the mean squares of the model (which in the case of a one parameter model like ours is equal to the sum of squares of the model, *SS*_*model*_) and the mean squares of the residual, obtained by dividing the sum of squares of the residual, *SS*_*residual*_, by the degrees of freedom, *df*. The threshold *F* value used to determine hits was 99.0 (*p* <. 01). A simulation generating an *F* value greater than 99.0 was recorded as a *hit*; an *F* value less than 99.0 was considered a *miss*. How reliably a syllable parse captures the data was assessed over multiple runs of the simulation in the form of a *hit rate*, defined as the number of hits divided by the total number of simulation runs.

Our choice of goodness of fit metric emphasizes the relationship between RSD values as opposed to the exact RSD values in the data by tolerating affine transformation between experimental RSD values and simulated RSD values. For example, consider a set of experimental RSDs such as LE-A = 20.5%, CC-A = 9.7%, RE-A = 5.1% reported for the Moroccan Arabic dyad *bal*~*dbal*. Simulated RSDs that are identical (20.5%, 9.7%, 5.1%) would of course provide a perfect fit to this data but so too would values that are linearly transformed, such as LE-A = 16.4%, CC-A = 7.76%, RE-A = 4.08%, which are multiplicatively related to the *bal~dbal* by a factor of. 8. Simulated RSD values of LE-A = 28.5%, CC-A = 17.7%, RE-A = 13.01%, which are shifted up from the data by a constant value of 8% would also provide a perfect fit to the data. Transformations such as these (additive, multiplicative) preserve the relationship between RSD values in affine space. Computing model error from a linear fit to experimental and simulated RSD coordinates therefore deemphasizes exact values in favour of magnitude relationships between intervals, i.e., the size of the difference between the RSD of the RE-A and LE-A intervals relative to the size of the difference between the RSD of the LE-A and CC-A intervals. This is highly appropriate for our aim of assessing which coordination topology underlies the data.

We highlight the key components of the fitting process. First, the fit of a coordination topology to data is evaluated on the basis of relationships between interval stabilities for all three relevant intervals. The fitting process assesses numerical predictions for each of the three intervals simultaneously. This is crucial because, as we will see, it is the pattern between all three interval RSDs that is needed to reliably assess syllable structure. In this respect, our approach contrasts with statements of inequality involving just two intervals, e.g., RE-A stability lower than CC-A stability implies simplex onset organization, used in past work. Second, we count a given simulation run as a *hit* as long as the model is above criterion with some value of anchor variability. This approach allows us to abstract away from gradient goodness of fit measures which could be sensitive to exact values of non-essential parameters and focus on our central theoretical question. Our notion of *hit rate* has a conceptual antecedent in other work in probabilistic grammar. It plays a similar role in model evaluation as the confidence scores employed in Albright & Hayes [75] and as the posterior probabilities of Bayesian models [1,76]. The probabilistic rules of English past tense formation developed in Albright and Hayes [75] are associated with a *raw confidence* score. Defined as the ratio of the number of times that a particular rule applies, the rule’s *hits*, by the number of times in which the environment for the rule is present in the data, the rule’s *scope*, the confidence score reflects the likelihood that the rule applies when its environment is met. In the case at hand, that of syllable structure, the *hit rate* proposed above provides a simple statistic summarizing the probability that the data was generated under the hypothesized syllable structure. A final key component of the modelling paradigm is the stochastic component, introduced in error terms associated with gestural landmarks and scaled in the case of anchor variability. Parameterizing discrete representations (coordination topology) via anchor variability allows us to reveal the range of RSD patterns consistent with simplex and complex onset syllables.

### Arabic

We start with data from a single speaker, reported in [13] and summarized in Fig 6. Table 1 shows interval RSDs and model hit rates for seven word sets (matched dyads and triads). RSDs for all word sets show the expected pattern of RE-A interval stability. The RE-A interval has a lower RSD than the CC-A interval and the LE-A interval. Model simulations were run following the procedure described earlier. The hit rates for the two models clearly reveal the superior performance of the simplex onset model in fitting the Arabic data. The simplex onset model achieved a significant fit to the data on at least 847 out of 1000 runs of the simulation and an average hit rate of 95.3%. The complex onset model achieved at most 4 hits out of 1000 trials and an average hit rate of 00.1%. The simplex onset model clearly outperforms the complex onset model.

**Table 1:** The relative standard deviation (RSD) of three intervals, left edge to anchor (LE-A), center to anchor (CC-A), and right edge to anchor (RE-A), for different Moroccan Arabic word sets and model hit rates for each syllabic organization, simplex onsets and complex onsets.

Word dyad / triad	Interval stability (RSD)			Hit rate	
	LE-A	CC-A	RE-A	Simplex onset	Complex onset
*dal~dbal*	20.5%	9.7%	5.1%	98.7%	00.3%
*tab~ktab*	6.8%	5.7%	5.5%	97.1%	00.4%
*lih~glih*	18.5%	10.7%	2.7%	98.3%	00.1%
*bati~sbati*	19.3%	7.1%	5.2%	84.7%	00.1%
*bula~sbula*	22.0%	11.1%	7.3%	88.5%	00.0%
*bulha~sbulha~ksbulha*	24.6%	15.9%	11.2%	100%	00.1%
*dulha~kdulha~bkdulha*	28.5%	22.3%	20.3%	99.9%	00.0%
	**Average hit rate**			**95.3%**	**00.1%**

We now turn to datasets drawn from the same speaker for which interval stability measurements are not always consistent with the phonetic heuristic for simplex onsets, that is, with RE-A stability as exemplified in Fig 1. Table 2 shows two sets of interval RSDs, for each word dyad or triad. The intervals for these word dyads or triads were quantified using the two measurement techniques, once with the C^Max^ anchor and once with the V^End^ anchor. When using the C^Max^ anchor, the RE-A interval showed lower relative standard deviation (RSD) than the CC-A interval. This is the canonical result for Arabic and, as with the data in Table 1, it goes along with theoretical evidence supporting the simplex onset hypothesis for Moroccan Arabic [42]. However, when the data were quantified using the V^End^ anchor the inverse stability pattern was found. This latter pattern is the same as that seen in English and would seem to support the complex onset hypothesis. In sum, in one subset of measurements it is the RE-A that is most stable, but in a different subset it is the CC-A interval that is most stable. We refer to cases of this sort as “stability reversals”.

**Table 2:** The relative standard deviation (RSD) of three intervals, left edge to anchor (LE-A), center to anchor (CC-A), and right edge to anchor (RE-A), calculated over Moroccan Arabic word sets using different landmarks, V^End^ and C^Max^, to right-delimit the intervals.

Word dyads / triads	Anchor	Interval stability (RSD)			Variability Index
		LE-A	CC-A	RE-A	
bulha~sbulha~ ksbulha	C^Max^	24.6%	15.9%	**11.2%**	22
	V^End^	23.9%	**17.8%**	18.2%	41
bal~dbal	C^Max^	20.5%	9.7%	**5.1%**	15
	V^End^	27.5%	**22.7%**	25.3%	63
tab~ktab	C^Max^	6.8%	5.7%	**5.5%**	14
	V^End^	12.2%	**7.7%**	10.0%	26
bula~sbula	C^Max^	22.0%	11.1%	**7.3%**	19
	V^End^	14.6%	**6.5%**	6.9%	26

The bold values indicate the intervals with the lowest RSD. For intervals right-delimited by the C^Max^ landmark, the RE-A interval has the lowest RSD. For intervals right-delimited by the V^End^ landmark, the CC-A interval has the lowest RSD. The rightmost column provides the standard deviation of the RE-A as an index of variability for the corresponding word set.

One response to such inconsistencies would be to conclude that temporal stability indices are unreliable in diagnosing syllabic organization (“everything goes”) or even that syllable structure does not and need not, as Kahn ([7] pages 16–17) asserted, have consistent phonetic indices. A different approach is to appreciate that the relation between abstract phonological organization and these indices may not be straightforward, and undertake the task of developing tools enabling the systematic study of this relation. More generally, the problem met here is a specific instance of a larger problem in present day cognitive science, namely, the problem of evaluating qualitative theoretical constructs with variable experimental data.

We illustrate our approach in two steps. First, we show that it is possible to evaluate syllabic organization even when phonetic heuristics produce ambiguous or misleading results, as in the case of the stability reversals in Table 2. In a second step, we use our models to make explicit the relation between theoretically posited syllable parses and the entire range of their quantitative consequences. The models will be employed as analytical tools to study the effect of variability on indices of temporal stability. Via the models we generate simulated data. The focus will be on how the patterning of temporal stability indices changes as we change variability in the data which, it will be recalled, is done in our simulations by systematically changing anchor variability.

We begin by applying our procedure for quantitative evaluation to the Moroccan Arabic word sets shown in Table 2. For each set of RSD values showing CC-A stability in Table 2, we again conducted 1000 simulations of each syllabic organization and evaluated the goodness of fit between simulated data and experimental data, as above. The hit rates for each case are given in Table 3. For the measurements under consideration, RSD of intervals right-delimited by the V^End^ anchor, the CC-A interval has a lower RSD than the RE-A interval and the LE-A interval. Nevertheless, the simplex onset model outperforms the complex onset model in each case.

**Table 3:** Hit rates for each syllabic organization, the simplex onset model and the complex onset model, for sets of Moroccan Arabic words that show stability reversals.

Word dyads / triads	Anchor	Hit rate	
		Simplex onset	Complex onset
bulha~sbulha~ksbulha	V^End^	85.4%	00.7%
bal~dbal	V^End^	45.9%	01.8%
tab~ktab	V^End^	52.2%	02.8%
bula~sbula	V^End^	86.0%	00.6%
	**Average hit rate**	**67.4%**	**1.5%**

**Fig 7:**
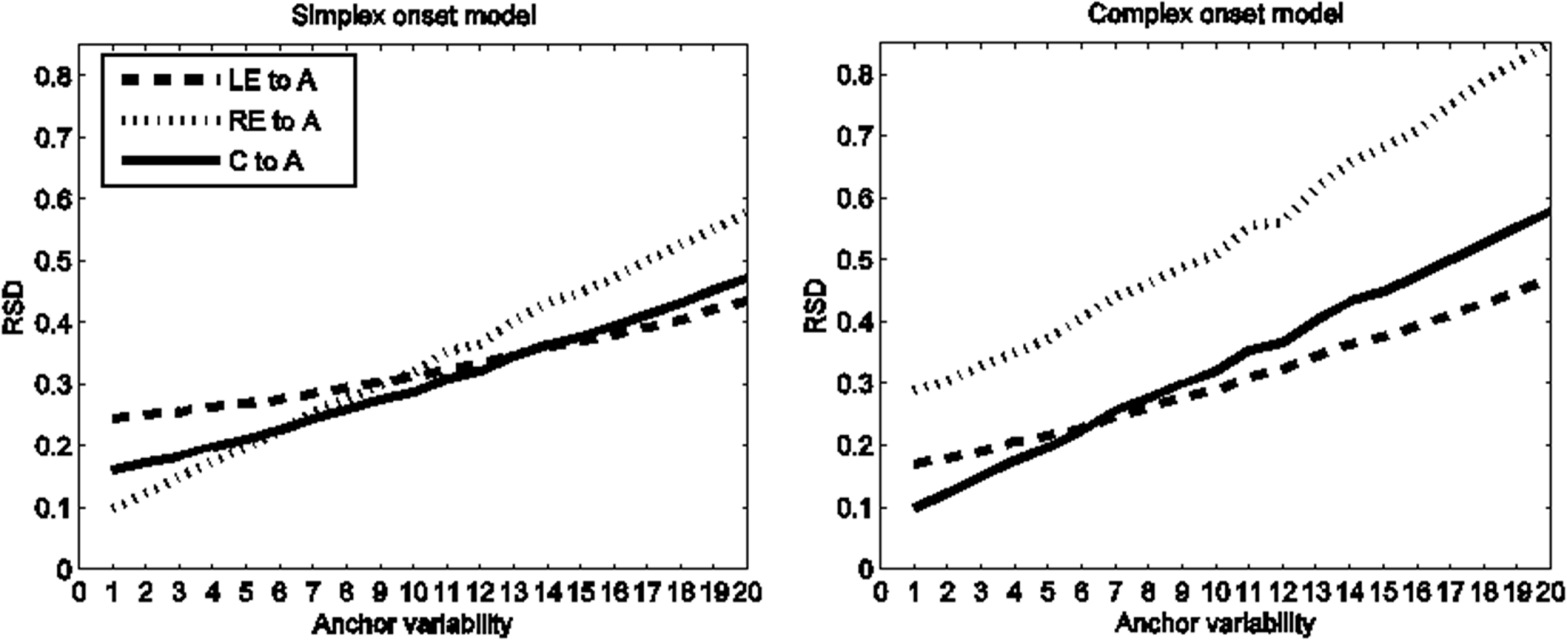
Simulation results for the simplex onset model (left) and the complex onset model (right). The *y*-axis shows the RSD of the LE-A, RE-A and CC-A intervals. The *x*-axis shows anchors from lowest to highest variability (1 to 20). For anchors of low variability, anchors 1–6, the RE-A interval has the lowest RSD for the simplex onset model (left) and the CC-A interval has the lowest RSD for the complex onset model (right). Beyond anchor 7, however, stability patterns, expressed in terms of inequalities, change. For the simplex onset model (left), the RE-A interval becomes more variable than the CC-A interval; for the complex onset model (right), the CC-A interval becomes more variable than the LE-A interval. These changes in patterns of RSD inequalities obscure the expected phonetic consequences of the underlying syllabic structure. The main point illustrated is that the mapping between abstract syllabic organization and phonetic stability patterns cannot be expressed in terms of canonical or invariant stability patterns. The same symbolic organization, e.g. that of simplex onsets, surfaces with the expected phonetics of simplex onsets for one range of anchor values (1–6) but also with the expected phonetics of complex onsets for another range of parameter values (anchor value 7 and beyond).

Given that this subset of Arabic data shows CC-A stability and that CC-A stability has been considered prototypical of complex onset organization (see Fig 1, right), why does the simplex onset model outperform the complex onset model? Through simulation, our models allow us to sharpen reasoning about the relation between syllable structure and continuous indices of that structure in articulatory data. As the non-essential variable (anchor variability) is scaled, the interval RSDs change in accordance to the structure dictated by the essential variable (coordination topology). The result is a pattern of change, or dynamic, that characterizes any given syllabic structure (the essential, qualitative form) as a function of scaling or changing the non-essential variable in the model. The dynamics of interval RSDs as a function of anchor variability are illustrated in Fig 7 for simplex onset (left) and complex onset (right) syllables. The lines show the evolution of the RSD, *y*-axis, of three intervals (LE-A, CC-A, RE-A) as a function of increasing anchor variability, *x*-axis. As anchor variability increases, the RSD of all three intervals increases. However, the different intervals increase at different rates. At low levels of the non-essential parameter (anchor variability), the two syllable structures impart different patterns of RSDs on the intervals, which, when expressed in terms of inequalities, reflect the expectations for each syllable type represented by ‘canonical’ temporal schemes as depicted in Fig 2. For simplex onset syllables, the RSD of the RE-A interval is lower than the CC-A interval and LE-A interval. For complex onset syllables, the RSD of the CC-A interval is lower than the RSD of the RE-A interval and LE-A interval. These are the two canonical stability patterns assumed to characterize simplex and complex onsets; see Fig 2, left and right, respectively. But as anchor variability increases, the RSD of the RE-A interval increases at a faster rate than the RSD of the CC-A interval. A crossover point can thus be seen for simplex onset syllables after which the CC-A interval emerges as having better stability (lower RSD) than the RE-A interval. The stability pattern has changed. Specifically, it has changed to an English-like pattern expected for languages instantiating the complex onset hypothesis, even though the model generating the data here embodies the simplex onset hypothesis.

The model simulations permit one to see that there are stringent conditions for the occurrence of each stability pattern. Both stability patterns (RE-A more/less stable than CC-A) are consistent with simplex onset organization. Given a corpus and two sets of intervals delimited by different anchors extracted from this corpus, the model embodying simplex onsets predicts the following implicational relationship: if one set of intervals shows CC-A stability and the other shows RE-A stability, then the former set of intervals must correspond to an anchor with higher variability than the later. The opposite relationship is precluded; it is not the case that “everything goes”.

Such predictions allow us to better diagnose syllable structure in the phonetic record. For simplex onsets, it is only under such conditions of higher variability where the CC-A interval may be found to show a stability advantage over the RE-A interval. Returning to Table 2, the rightmost column reports a variability index, the standard deviation of the RE-A interval. This index for intervals right-delimited by the V^End^ anchor is higher than for the corresponding intervals right-delimited by the C^Max^ anchor. The key point is that, as predicted above, the sets of measurements showing CC-A stability (lower RSD for the CC-A interval than for the RE-A interval) have a higher variability index than the sets of measurements showing RE-A stability. The model hits reported in Table 3 add quantitative detail to these qualitative predictions. On the subset of Arabic data showing CC-A stability (Table 2), the simplex onset model outperforms the complex onset model because the interval stabilities predicted by the simplex onset model are quantitatively closer to the stabilities in the experimental data than those predicted by the complex onset model. We can thus see that although CC-A stability has been viewed as a phonetic index of complex syllable onsets [9,10,13,31,63], CC-A stability does not necessarily implicate complex onset organization. More generally, the simulations in Fig 7 demonstrate that the mapping between intended syllable structure and stability patterns cannot be expressed coarsely in terms of invariant stability patterns. The simplex onset model is consistent with both RE-A stability and CC-A stability. The stability pattern changes and specifically it changes systematically as a function of anchor variability. This fact reveals the fallibility of diagnosing syllabic organization via RSD patterns expressed in terms of static inequalities. As we have illustrated by the model-experimental data fits in Table 3, our models go further because they correctly diagnose syllabic organization and make sense of the seemingly inconsistent results concerning stability reversals in Table 2.

Before moving on to data from a language admitting complex onsets, we next consider a larger dataset for which variability is contributed not by the measurement method (C^Max^ anchor versus V^End^ anchor) but by pooling data across four Arabic speakers. In other words, in this dataset rather than calculating RSDs separately for each speaker, as is typically done to reduce variability, we have calculated the RSDs across speakers. Resulting RSDs and hit rates for the simplex and complex onset model on this dataset are in Table 4. Because interval measurements now incorporate inter-speaker variation in addition to the other sources of variability, the RSDs are quite a bit higher than in the single speaker data discussed above, particularly for the RE-A interval and the CC-A interval (the intervals in this dataset were all right-delimited by the C^Max^ anchor). For two of the three word sets, *lan~flan~kflan* and *kulha~skulha~mskulha*, the CC-A interval has a lower RSD than the RE-A interval. Despite CC-A interval stability, the simplex onset model again outperforms the complex onset model on Arabic data. In short, the stochastic models are not misled by inter-speaker variability just as they were not misled by measurement variability in the single speaker data.

**Table 4:** The RSD of three intervals, left edge to anchor (LE-A), center to anchor (CC-A), right edge to anchor (RE-A) calculated across multiple (10–18) repetitions by four speakers of Moroccan Arabic.

Word triads	repetitions	speakers	Interval stability (RSD)			Hit rate	
			LE-A	CC-A	RE-A	Simplex onset	Complex onset
lan~flan~kflan	10–18	4	32.8%	**26.8%**	26.9%	82.4%	00.0%
kulha~skulha~mskulha	10–18	4	32.8%	**26.6%**	30.2%	54.5%	00.0%
bulha~sbulha~ksbulha	10–18	4	27.1%	25.0%	**24.7%**	90.2%	00.0%
			**Average hit rate**			**75.5%**	**00.0%**

The RSD of the CC interval is lower than the LE and RE intervals for two of the three word sets. However, for all word sets, the simplex onset model achieves a greater hit rate than the complex onset model.

In sum, the simplex onset model outperforms the complex onset model on Arabic data. Moreover, it does so in pockets of Arabic data showing CC-A interval stability due to either measurement variability (Table 2 and Table 3) or inter-speaker differences (Table 4). The modelling paradigm sees through these sources of variation to reveal phonological organization in terms of syllable structure. The improved precision of our technique, over the use of heuristics based on stability inequalities, derives from two sources. First, our models expose how coordination topologies, the essential variable in our approach, structure relationships between interval stabilities. Second, the details of our fitting process, which takes into account the RSD of all three intervals and the relationship between them, is sensitive enough to capture in the data structure imparted by phonological variables in the model. We have shown that both simplex and complex syllabic structures may generate patterns whereby the CC-A interval is more stable than the RE-A interval. However, the fine-grained relationships amongst stabilities in the data (at levels of variability where both syllable parses would predict CC-A interval stability) are more consistent with the simplex onset model than the complex onset model. The stochastic interpretation of phonological structure proposed in our approach thereby succeeds in adjudicating between competing hypotheses when phonetic heuristics are ambiguous or misleading.

### English

We now ask whether a model embodying the complex onset hypothesis would outperform a model embodying the simplex onset hypothesis for data drawn from a language admitting complex onsets (the reverse of what we met above for Arabic). It is generally accepted that English is such a language [7]. As reviewed earlier, it is standard to assume that strings such as /kru/ ‘crew’ are parsed into a single syllable in English, with a complex two-consonant cluster as the onset of that syllable. We predict that for English data, a model embodying the complex onset hypothesis (Fig 3, H^2^) would outperform a model embodying the simplex onset hypothesis (Fig 3, H^1^).

There are by now a considerable number of experimental studies on syllable structure and timing in English (see references in Section 1). As expounded above, a key component of our modelling paradigm is that it allows us to study the *relation* between structurally relevant intervals. This requires a complete set of measurements from the data. Some of the relevant work on English has focused on patterns of inequality between just two of the intervals. We model experimental data here for which a complete set of measurements are available. Since English is a better-studied language than Arabic, data are available from a larger number of speakers. There is a tradeoff, however, in the type of consonant clusters that are available. Our Arabic datasets included tri-consonantal clusters with both rising and falling sonority profiles. English is more limited in the range of clusters it allows.

Our first English dataset draws from work of Browman and Goldstein reported in [9]. This was the first study to use fleshpoints on articulatory organs to investigate the relation between temporal stability and syllable structure. This study provided over the word set [pɔt], [sɔt], [lɔt], [spɔt], [splɔt], [plɔt] measurements of the stability of all three relevant temporal intervals, LE-A, RE-A, and CC-A. Interval stability was reported in terms of the standard deviation of each interval calculated across the word set. In order to make the measurements directly comparable to those for Moroccan Arabic discussed in the previous sections, the relative standard deviation (RSD) of the English productions was calculated by dividing the standard deviation of each interval by the mean of that interval. Alongside this dataset, we analyzed a similar word set, *pend*~*spend* from another American English speaker collected using the EMA facilities at the University of Potsdam speech production lab. The reason for including this additional word set will become apparent later in the discussion.

**Fig 8:**
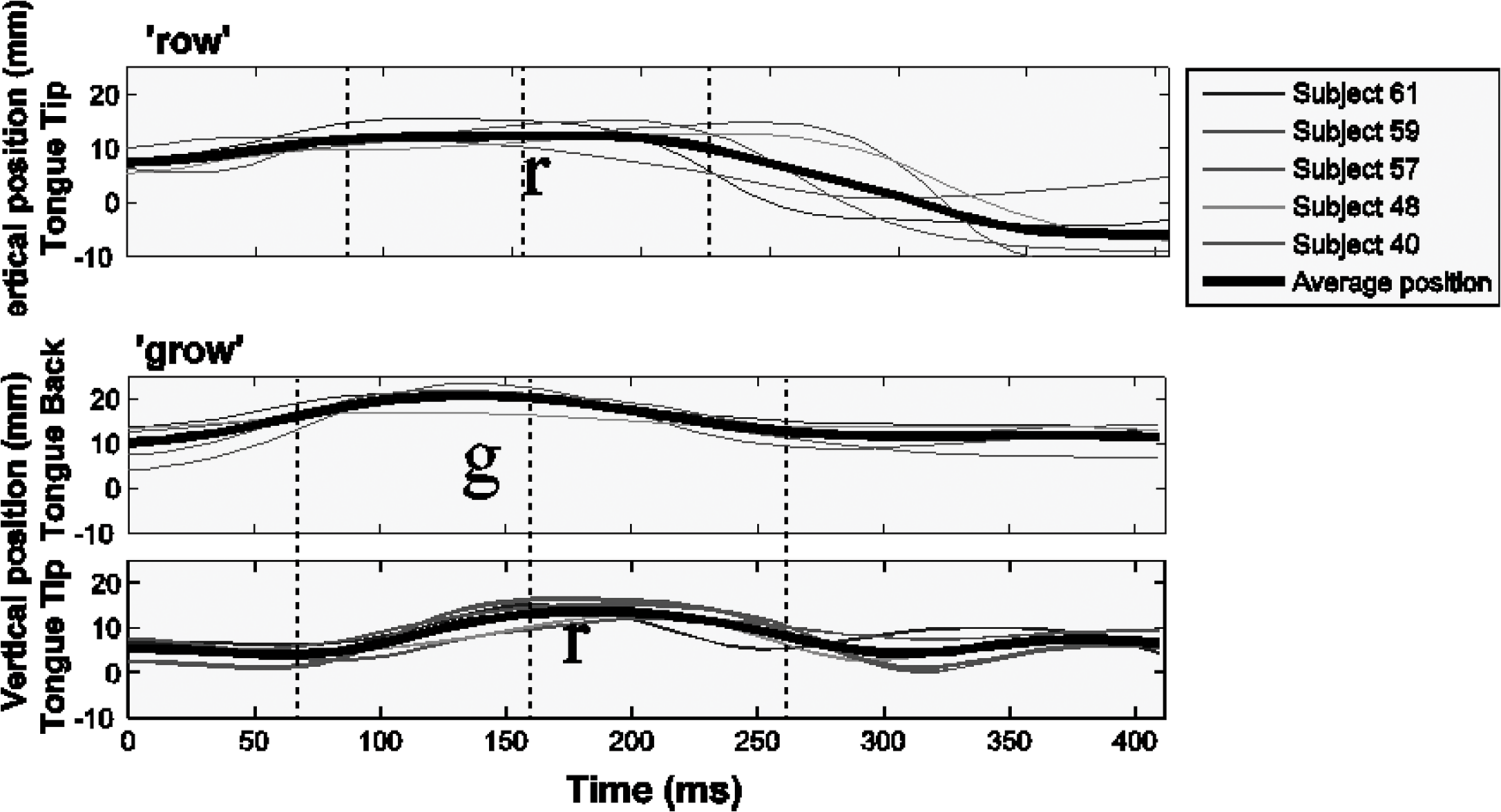
Articulatory recordings of English. The top panel shows the movement of the tongue tip during the production of ‘row’ by five speakers. The bottom two panels show the tongue tip and tongue back movement during the production of ‘grow’ by the same five speakers. The shading of the lines indicates the different speakers. The thick black line shows the average trajectory across speakers. Dotted vertical lines indicate the landmarks that left-delimit the temporal intervals of interest: left edge, center, and right edge. The movement trajectory of the tongue tip is shifted (to the right) in *row* relative to *grow* while the center landmark remains constant across words.

Our main source of English data was drawn from the Wisconsin X-ray microbeam speech production database [61]. This database contains recordings of a variety of tasks including production of sentences, passages and word lists from fifty-seven speakers of American English. Although not all speakers contributed recordings for all tasks and some recordings have missing data which make them unusable for our analysis, the Wisconsin datasets remain an archive of articulatory data that is extremely impressive in size. To illustrate how consonant clusters are timed relative to singleton consonant onsets in English, Fig 8 shows movement trajectories for the tongue tip (referred to as T1 in the database) for the word ‘row’ (top panel) and for the tongue tip and tongue back (referred to as T4) for the word ‘grows’ (bottom panel). These words were extracted from read sentences. The word ‘grows’ was extracted from the sentence “The noise problem grows more annoying each day” of Task 57 ([61] page 194). The word ‘row’ was extracted from the sentence “Things in a row provide a sense of order” of Task 56 ([61] page 192). Data from five speakers who contributed a complete set of measurements for both tasks 56 and 57 are shown in Fig 8. The dotted lines correspond to the landmarks introduced in Fig 1, namely, the left edge, center, and right edge. The key observation is that the center to anchor interval remains relatively constant across *row* and *grow*, while the left edge to anchor interval increases and the right edge to anchor interval decreases. This pattern can be contrasted with Arabic (Fig 2, Fig 5) where additional segments, e.g., adding [f] to [lan] (Fig 5) lengthen the left edge to anchor interval and center to anchor interval while leaving the right edge to anchor interval unperturbed. For the purposes of evaluating our models, we included additional speakers producing one or both of these words. A total of 25 tokens of *row* and 25 tokens of *grows* were extracted for analysis. These 50 tokens were drawn from 33 different speakers (some speakers did not produce data for both words) providing us with a high level of inter-speaker variability. As demonstrated for the Arabic data, our models are able to handle variability from multiple sources including (at least) differences across speakers and measurements. The *row-grows* dataset from English allows to ask whether the same holds true in a language with complex onsets.

The English datasets above concern word-initial clusters. No previous work on cluster timing in English has examined word-medial clusters. If the timing patterns assessed in our models are characteristic of syllable-initial as opposed to just word-initial clusters, then the same patterns should be also met in word-medial clusters, since in both word positions the syllabification is claimed to be the same. We have pursued this prediction and thus extended the empirical range of the correspondence between timing and syllables, by examining a set of word-medial clusters from the Wisconsin X-ray microbeam speech production database [61], obtaining articulatory measurements for a set of words from a population of 21 to 40 speakers, depending on the word quantified, as described below. The number of words and speakers were determined by our quantification requirements. Specifically, we looked for words with word-medial syllable onset clusters for which the consonantal gestures for all the onset consonants and the postvocalic consonant could be measured (often single gestures could not be measured for one or the other speaker). Furthermore, we chose a set of words with uniform prosodic structure, specifically with stress on the second syllable, because in bisyllabic words the position of stress is known to affect speakers’ judgments about syllabification [77,78]. Within this archive, we converged on six words, three with single C onsets and three with CC onsets: be[f]ore, u[p]on a[b]out, be[tw]een, a[cr]oss, hi[sp]anic, with 21–40 repetitions per word. A few other two syllable words in the corpus are stressed on their second syllable, but these words appeared in contexts which made it impossible to measure the gestures for the majority of speakers. The speakers were not the same for every word because at times a gesture could not be measured for a speaker in one word, but all gestures could be measured in another word produced by the same speaker. In the organization of the Wisconsin database, each word occurred in a specific task number. The S1 File lists subject codes for the subjects whose data were measured for any given word as well as the tasks from which these words were obtained.

**Fig 9:**
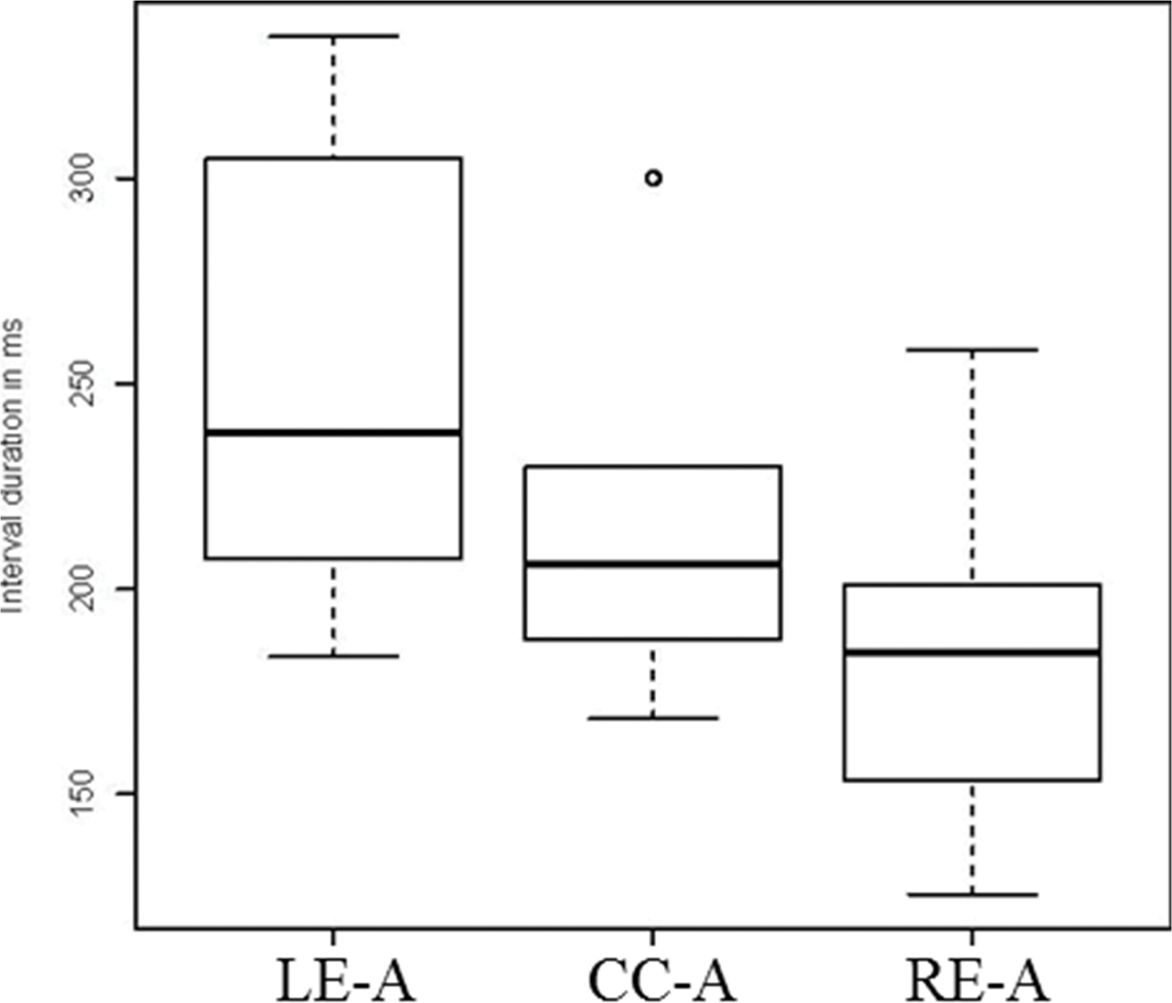
Duration of measured intervals in English. Boxes summarize mean duration calculated over 255 data points from 21–40 speakers (depending on the word) of each interval. Left bar: LE-A (left edge to anchor interval), middle bar: CC-A (center to anchor interval), right bar: RE-A (right edge to anchor interval).

Quantification of our data proceeded as follows. For every instance of our words, the left edge to anchor (LE-A), center to anchor (CC-A), and right edge to anchor (RE-A) intervals were measured following the procedures in Section 3.2. Over the productions of one word (e.g. 32 productions of *before*, 40 productions of *upon* and so on) mean interval durations were calculated. Fig 9 shows the means for the three interval durations across the different words. The boxes are based on six values each, i.e. one value per word. Relative standard deviations (RSD) were calculated by dividing the standard deviation over all six values of an interval by the corresponding interval mean. Resulting RSD values are summarized in Table 5. For the word-medial dataset, they are as follows: LE-A: 23%, CC-A: 21%, RE-A: 24%. The RSD is therefore lowest for the CC-A interval, suggesting complex onset organization for the English word-medial onsets examined here.

**Table 5:** The mean, standard deviation, and relative standard deviation of three intervals, left edge to anchor (LE-A), center to anchor (CC-A), right edge to anchor (RE-A), calculated across four English word sets with varying numbers of speakers.

Dataset		Interval stability (RSD)			Hit rate	
	Speakers	LE-A	CC-A	RE-A	Simplex onset	Complex onset
*pot~sot~spot~lot~plot~splot*	1	14%	8%	23%	53.7%	93.0%
*pend~spend*	1	8%	12%	19%	40.7%	93.6%
*row~grows*	33	13%	13%	17%	74.3%	98.2%
*be[fore]*,*u[pon] a[bout]*, *be[tween]*, *a[cross]*, *hi[spanic]*	Varies by word from 21–40 (see S1 File)	23%	21%	24%	41.1%	99.6%
		**Average hit rate**			**52.5%**	**96.1%**

The hit rates for the simplex onset model and the complex onset model are given in the right two columns. For each word set, the complex onset model provides a higher hit rate than the simplex onset model.

We now turn to report model hit rates for our English datasets described above. Following the same methodology as for the Arabic data, simulations with the simplex and complex onset models generated RSD values that were evaluated against the RSD values of the three intervals of interest in the English data. That is, the RSDs from the experimental data were compared to values output from model simulations based on a simplex onset parse, e.g., [sp.lɔt]~[p.lɔt]~[lɔt], and a complex onset parse, e.g., [splɔt]~[plɔt]~[lɔt], of the target strings. Anchor variability was increased by 5ms across 15 steps, providing a range of variability from 0 ms (anchor 1) to 70 ms (anchor 15). The phonetic parameters in the model, consonant duration, inter-plateau interval, and vowel duration were set to means in the data, as was done for Arabic. For dyads such as *pend~spend*, word replicas beginning with one and two initial consonants were simulated. For the Browman & Goldstein data, which also includes the tri-consonantal cluster *spl* in *splot*, word replicas beginning with one, two and three initial consonants were simulated. The simulated words were generated based on a value for the essential variable (syllable structure) and a range of values of the non-essential variable (anchor index).

The average hit rate reported in Table 5 for the complex onset parse was 96.1% compared to 52.5% for the simplex onset parse. This indicates, in line with our expectations, that the complex onset parse provides a better fit to this data than the simplex onset parse. Examining the interval stability patterns for the individual datasets in Table 5, we see that, in some (but not all) cases, the RSD of the CC-A interval is lower than the RSD of the RE-A and LE-A intervals. This pattern of CC-A interval stability has typically been taken to indicate organization of consonant clusters into complex syllable onsets (Fig 1, right). However, we have also seen that the simplex onset model is consistent with this pattern of RSDs (Fig 7). Specifically, as variability is increased, the pattern output by the simplex onset model goes from RE-A < CC-A to CC-A < RE-A. The RSD pattern output by the complex onset model is CC-A < RE-A at low levels of variability. Thus, at high levels of variability, the simplex onset model can mimic the variability pattern (expressed in terms of inequalities) of the complex onset model. Nevertheless, as seen in the hit rates reported in Table 5, on English data the complex onset model outperforms the simplex onset model. The result is due to the way that interval RSDs are uniquely structured by the distinct coordination topologies of the simplex and complex onset models.

Besides those word sets for which the CC-A interval was more stable than the RE-A and LE-A intervals, Table 5 also reports results for two cases in which the LE-A interval was the most stable (had the lowest RSD) of the three intervals. These come from the *pend~spend* and *row~grows* dyads. Minimal stability for those is found neither for the CC-A nor for the RE-A intervals, but rather for the LE-A interval. These cases are not amenable to a syllabic diagnosis in terms of the inequality relations that have been proposed, e.g. *center to anchor lower than left or right edge to anchor stability*. In absence of our modelling paradigm, this pattern of stability would not allow one to distinguish between syllable parses. For the same reason that our approach can distinguish between cases of CC-A stability indicative of simplex onsets (as we saw in Arabic) and cases of CC-A stability indicative of complex onsets (as in the English data), it succeeded in distinguishing between syllable parses for the dataset that shows LE-A interval stability. In terms of stability inequalities, both the simplex and the complex onset model predict LE-A stability at high levels of variability (see Fig 7). Crucially, the models predict relations between interval RSDs that are more precise than statements of inequalities and these relations uniquely differentiate syllabic structure. This level of precision is necessary to differentiate parses of our *pend~spend* and *row~grows* dyads.

We have therefore seen that the complex onset model outperforms the simplex onset model on English data. For Arabic data, the reverse is true. The simplex onset model outperforms the complex onset model. The models make the right predictions for these languages and they do so even when the phonetic heuristics break down.

Before closing, we wish to scrutinize an aspect of our results which points to the presence of additional factors shaping the experimental data to model fits. When we look at the hit rates of our models on the two languages, we see that the margin by which the simplex onset model outperforms the complex onset model on Arabic data is substantially greater than the margin by which the complex onset model outperforms the simplex onset model on English data. The best performance of the complex onset model on Arabic data was a hit rate of 2.8% on the *tab~ktab* dyad. In contrast, the simplex onset model achieved a hit rate of 74.3% for the *row~grows* data in the X-ray microbeam corpus.

Due to design characteristics of the X-ray microbeam corpus, the *row~grows* dataset involves data drawn from 33 different speakers, each contributing one or two instances of these words. Typically, in our Arabic datasets, fewer speakers, e.g. up to 4, produce more, e.g. up to 18, iterations of words (see Table 4). Thus, before we identify the source of this asymmetry, we evaluate the extent to which the degree of inter-speaker variability in the English data may have contributed to it. To do so, we sub-divided the *row~grows* word set, our largest English word set, into mini-corpora each with a lower level of variability than the aggregate word set. Specifically, the *row*~*grows* corpus of 50 tokens from 33 speakers reported in Table 5 was divided into smaller corpora of 8 tokens each (4 tokens of *row* and 4 tokens of *grows*). The divisions were made by ordering the 50 words of the larger dataset according to the duration of the CC-A interval. The first corpus contained the 4 tokens of *row* with the shortest CC-A interval and the 4 tokens of *grows* with the shortest CC-A interval. The second corpus contained the words with the next shortest interval and so on until 6 corpora of 8 tokens each were formed. Dividing the data in this way yielded a set of corpora each having a smaller degree of overall variability, as indexed by the standard deviation of the RE-A interval, than the larger corpus.

The interval statistics of the six mini-corpora together with hit rates from both syllable parses are shown in Table 6. Although the complex onset model outperforms the simplex onset model on all of the sub-corpora, the simplex onset model is also able to score a substantially larger number of hits on each sub-corpus than the complex onset model was able to achieve for any Arabic word set.

**Table 6:** Mean, standard deviation, and relative standard deviation of three intervals, left edge to anchor (LE-A), center to anchor (CC-A), right edge to anchor (RE-A), for 6 groups of 8 productions of *row* and *grows* by speakers of American English drawn from the X-Ray microbeam speech production database.

*row~grows*corpus subset		Interval statistics			Hit rate	
		LE-A	CC-A	RE-A	Simplex	Complex
1	Mean	240	212	180		
	SD	20	19	28		
	RSD	**8%**	**9%**	**16%**	92.1%	99.2%
2	Mean	278	240	204		
	SD	22	7	21		
	RSD	**8%**	**3%**	**10%**	46.9%	95.7%
3	Mean	287	254	220		
	SD	14	8	21		
	RSD	**5%**	**3%**	**10%**	56.3%	78.5%
4	Mean	316	273	229		
	SD	16	6	25		
	RSD	**5%**	**2%**	**11%**	52.6%	92.8%
5	Mean	328	290	247		
	SD	23	11	19		
	RSD	**7%**	**4%**	**8%**	39.3%	94.9%
6	Mean	337	302	266		
	SD	20	11	27		
	RSD	**6%**	**4%**	**10%**	62.5%	91.6%

The hit rates for the simplex and complex onset models are given in the right two columns.

The results in Table 6 show that even when we divide our largest corpus into word sets with lower overall variability, the hit rate asymmetry persists. This implies that the asymmetry we observed in hit rates is not due to inter-speaker variability. Rather, the asymmetrical hit rates seem to have their source in phonetic forces which arise only in the context of complex onsets. Specifically, this modelling result echoes the patterning of known phonological markedness relations of syllables. Both within and across languages, syllables with complex onsets are more marked than those with simplex onsets [6,44]; moreover, there is an implicational relation amongst syllable types such that languages with complex onset syllables also have syllables with simplex onsets while the reverse is not true [79]. The direction of the hit rate asymmetry in our results indicates that it may be harder to reliably parse complex onset syllable types from the phonetic signal, which could potentially be related to the markedness of these structures.

Returning to the temporal alignment schemas in Fig 1, it can be seen that the two different phonological organizations impose different phonetic demands as the string changes from CV to CCV. In particular, as we perturb the phonological sequence by adding a consonant, simplex onset organization (Fig 1, left) prescribes a *lengthening* of the CC-A and LE-A intervals and no change (stability) in the RE-A interval. As a segment is added to a CV to form CCV, e.g. *bul* to *sbul*, the CC-A interval is naturally lengthened by the increase in segmental content. Lengthening of an interval as a result of the addition of a segment meets no phonetic constraints. Notably, no interval is required to shorten under simplex onset organization. In contrast, complex onsets (Fig 1, right) prescribe a *shortening* of the RE-A interval (as well as a lengthening of the LE-A interval). As a segment is added to a CV to form CCV, e.g. *lay* to *play*, complex onset organization requires a decrease in the RE-A interval, which is coextensive with the acoustic portion of the vowel of the syllable. Apparently, the RE-A interval does shorten in our English data but not to the extent prescribed by the complex onset topology. The reason is possibly that RE-A shortening potentially risks the perceptual recoverability of the vowels (following the initial consonants) for some word sets. Thus, phonetic, functional pressures of perceptual recoverability, at play only in the complex onset organization, constrain the veridical actuation of temporal alignment schemas. In the presence of such phonetic constraints on temporal compression, the simplex onset model, aided by stochastic versions of timing relations, can fit the English data, although not nearly as well as the complex onset model. Despite the asymmetry, the complex onset model consistently outperformed the simplex onset in all our English data.

### Global evaluation of syllable-specific dynamics

We have so far reported hit rates in our results for particular word sets in Arabic and English. For such word sets, hit rates were obtained by evaluating experimental data against data simulated from pairs of model parameter values—one value for the essential variable (coordination topology: simplex vs. complex) and one for the non-essential variable (anchor variability). Our approach also allows us to evaluate syllabic organization over and above local fits to particular word sets. Specifically, in this section, we pursue an evaluation of model performance across different pairs of essential and non-essential parameter values, providing converging results with those reached in the preceding section.

In our approach, the phonetic values corresponding to a phonological organization are not fixed or static. Rather, the phonological organization prescribes a pattern of change in temporal stability indices as the non-essential variable is scaled (see Fig 7). In other words, each coordination topology generates a global stability profile. It is this more global characterization as opposed to individual stability values that can be seen as a signature of phonological organization. To examine our data also from this perspective, we have arranged individual word sets into pairings of phonetic indices (RSD values) and estimates of our non-essential variable values. Across the values of the non-essential variable found naturally in the data, we can compare changes in phonetic indices with the stability profiles generated by the simplex and complex models. This allows for a global assessment of how phonological organization may apply uniformly across markedly different segmental sequences.

**Fig 10:**
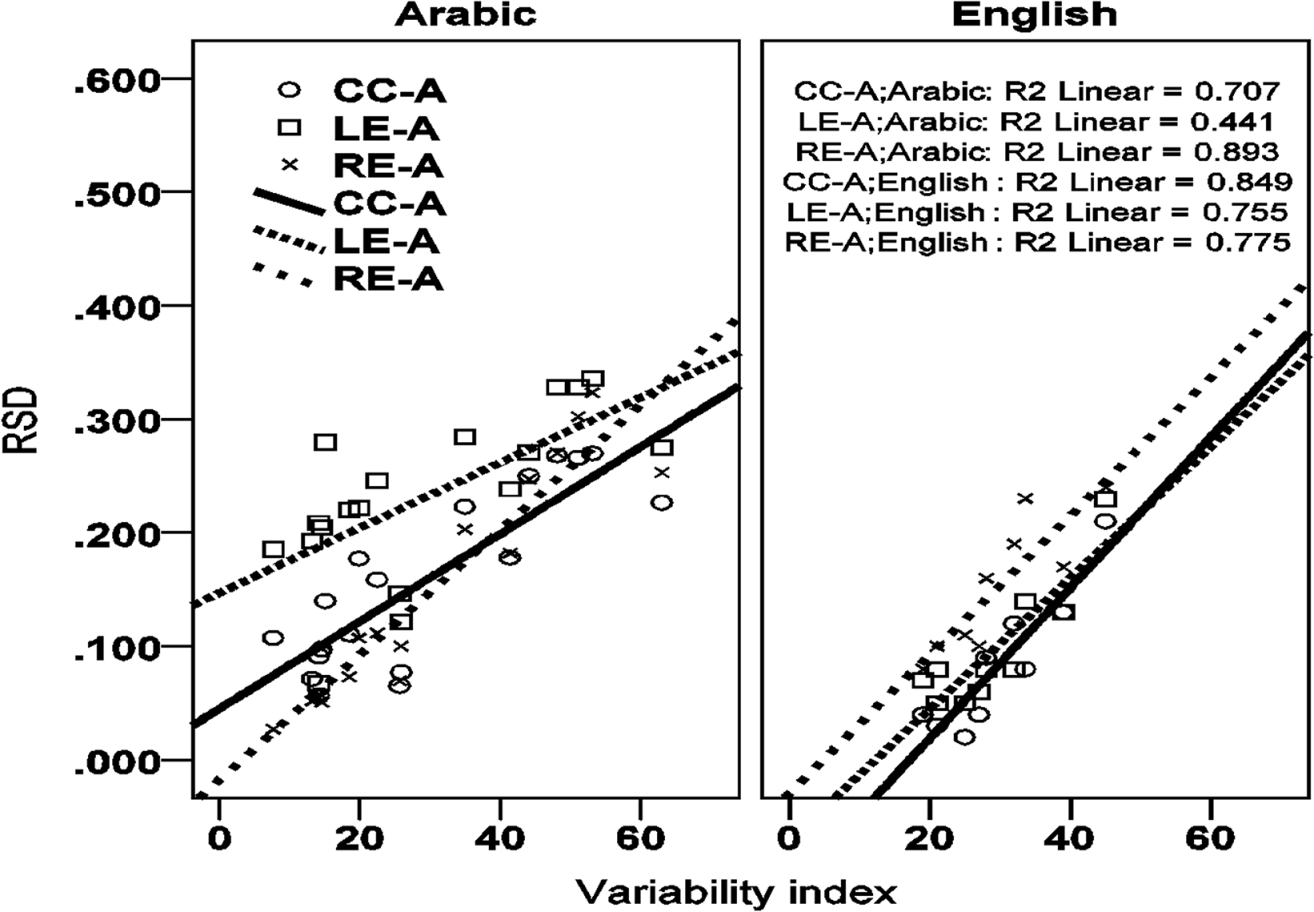
Interval stability dynamics for Arabic and English. Regression lines fit to RSDs of LE-A, CC-A, and RE-A intervals, y-axis, plotted against the standard deviation of the right edge to anchor interval for Arabic (left) and English (right) data. All RSDs reported in the paper for both languages are shown in the figure. The patterns in the regression lines for Arabic correspond to the simplex onset dynamic (Fig 7, left); the patterns for English correspond to the complex onset dynamic (Fig 7, right). Regression fits are significant at the *p* < .01 criterion for all intervals shown.

Fig 10 shows the interval data discussed in this paper (both Arabic, left, and English, right), including the sub-corpora in Table 6, re-presented as a function of our variability index (standard deviation of the right edge to anchor interval). The *y*-axis shows interval RSD and the *x*-axis shows the index of variability. The variability index is a significant predictor of RSD for all of the intervals (English, LE-A [*B* = .869, *t*(9) = 4.970, *p* <. 001]; English, CC-A [*B* = .921, *t*(9) = 6.699, *p* <. 001]; English, RE-A [*B* = .881, *t*(9) = 5.254, *p* < .001]; Arabic, LE-A [*B* = .664, *t*(17) = 3.55, *p* < .01]; Arabic, CC-A [*B* = .841, *t*(17) = 6.209, *p* < .001]; Arabic, RE-A [*B* = .945, *t*(17) = 11.545, *p* < .001]. The pattern in the regression lines can be compared to the dynamics of interval RSD predicted by our models (Fig 7). For the Arabic data (Fig 10, left), the dynamic of RSD change corresponds to the simplex onset model (Fig 7, left). At low levels of variability, the RE-A interval has a lower RSD than the CC-A interval and the LE-A interval. However, as variability increases, the RSD of the different intervals change at different rates. We can, therefore, observe a crossover point after which the CC-A interval has a lower RSD than the RE-A interval. The English pattern (Fig 10, right) is different. Despite the asymmetry found locally in hit rates for individual word sets, the phonetic indices of English syllables collectively compose a distinct dynamic. That dynamic differentiates English from Arabic and conforms to the predictions of complex onset topology when viewed through the lens of our non-essential variable. Here, as in the complex onset model simulations (Fig 7, right), the RSD of the RE-A interval is greater than the RSD of the CC-A interval and the LE-A interval across the board. At low levels of variability, it is the CC-A interval that has the lowest RSD. As variability increases, the LE-A interval becomes more stable (lower RSD) than the CC-A interval. This pattern is distinct from Arabic and it is different in just the way predicted by the models. The dynamic of RSD change for English corresponds to the simulations of the complex onset model.

The datasets plotted in Fig 10 naturally contain different levels of variability, owing to the different speakers, phonetic contexts, and words that have left their imprints on the measurements. Under the modelling approach we have developed here, controlling these sources of variability is not required for assessing syllable structure. On the contrary, the range of variability in the data we have modelled allows us to confirm that the hit rates achieved for individual word sets are part of the broader pattern, sketched in Fig 10 and predicted by the coordination topologies that structure syllable-specific dynamics of temporal stability. The two fitting approaches we have employed, the hit rates in section 4.2 and the stability profiles in this section, provide an inter-locking diagnosis of syllabic organization, converging on the same main result from different perspectives on the phonological dynamic.

### Overview and prospects

We have developed a modelling paradigm that allows us to differentiate syllable parses on the basis of experimental data tracking the movement of fleshpoints on speech organs. The central idea instantiated in our paradigm is that different modes of phonological organization dictate specific patterns of variability in the data. We have revealed the unique dynamics of simplex and complex onset syllables by scaling a non-essential variable in the model. Across values of the non-essential variable, patterns of change in the phonetic indices of syllables were structured by phonological form, the coordination topologies corresponding to simplex and complex onsets.

We used the models to differentiate syllable parses in two ways. First, we compared the phonetic indices (RSDs of syllable-referential intervals) of each word set from Arabic and English to the dynamics of each syllable parse (simplex, complex). Quantitative fitting produced a hit rate for each model-data pairing based upon some local area of the dynamic landscape. On Arabic data, the simplex onset model clearly out-performed the complex onset model, producing higher hit rates for all word sets regardless of the level of variability in the data. This is significant because variability is known to cause phonetic heuristics for simplex onsets to break down [55]. On English data, the complex onset model produced a higher hit rate than the simplex onset model. Our second method used model simulations more holistically to evaluate syllabic organization. The change in phonetic indices (as the non-essential variable is scaled) predicted by our models (Fig 7) was compared to change in phonetic indices found across the data (Fig 10). The variability index that functioned as non-essential variable in our model was a significant predictor of change in RSD values in the data. Moreover, the pattern of change in RSD values for English and Arabic was distinct and followed the pattern predicted by our models. Arabic conformed to the simplex onset dynamic. English conformed to the complex onset dynamic. This method of evaluating syllable structure makes use of the natural variability in the data and the entire landscape of our simulated dynamics.

Gaining a better understanding of the relation between abstract syllabic form and its phonetic manifestations holds significant potential long-term benefits for assessing normal language development as well as impairment or developmental delay in patients and child populations. Syllable structure has been implicated in the error patterns of patients with apraxia of speech [80] and may also play an important role in diagnosing reading disorders since developing the skill of reading also requires mastery of the relative timing of speech movements. In children characterized as ‘impaired’ readers, there is a close link between coordination and reading ability [81]. Carello et al. found evidence for this link persisting well after childhood by demonstrating correlations between motor coordination and reading ability in university undergraduates who would not normally be considered impaired [82]. As a factor dictating language-specific patterns of relative timing, syllable structure is thus crucially implicated in both speech as well as reading disorders. Although the main contribution in this work is the development of tools to rigorously evaluate the fit between competing syllabic hypotheses and articulatory patterns, being able to apply such tools using also acoustic data would increase impact significantly. This is because it would enable virtually unlimited and quick future access to languages and speaker populations for which articulatory data is difficult or impossible to obtain. At present, experimental sessions with advanced techniques such as EMA are not feasible with certain patient groups or young children. Acoustic data, however, are far easier to obtain. Moreover, while the techniques for tracking fleshpoints on speech articulators are becoming more widespread, they remain relatively expensive and highly time-consuming. Partly as a result of this, data acquisition and processing time dictate that most EMA studies report on only a small number of participants (less than 10).

With these considerations in mind, we have begun to explore the possibility of modelling acoustic data with the techniques developed here. Acoustic data provide indirect information about articulatory movement, but they can be acquired and processed quickly. Moreover, large acoustic corpora are already available for some languages. One example of such a corpus is AusTalk (https://austalk.edu.au/), which aims to collect audio-visual recordings of 1000 speakers of Australian English gathered from 10 different regions (Canberra, Sydney, Armidale, Darwin, Alice Springs, Brisbane, Adelaide, Hobart, Melbourne, Perth) across Australia. The AusTalk recordings are standardized in that the entire corpus was collected with the same equipment (12 identical, portable, self-contained recording stations) and materials, including word lists, sentences, read stories, and spontaneous speech [83].

To evaluate the feasibility of extending our modelling approach to acoustic data, we quantified the relevant temporal intervals, LE-A, CC-A, RE-A, in a subset of the AusTalk corpus. A total of 98 speakers, 9–10 speakers from each of the 10 regions of Australia represented in the corpus were selected at random for analysis. The corpus materials included three repetitions of the word *raw* and three repetitions of the word *draw*. In our acoustic measurements of these words, we sought landmarks that correspond as closely as possible to the articulatory events used to quantify intervals in the EMA and X-ray microbeam data. All intervals were right-delimited by a common anchor, the offset of acoustic energy in the first formant, an acoustic index of the V^end^ landmark. The intervals were left-delimited by landmarks that could be measured reliably in the acoustics: the LE-A interval was left-delimited by the onset of acoustic energy in the waveform. The RE-A interval was left-delimited by the offset of the immediately prevocalic consonant, as indicated in the case of *raw~draw* by an increase in F3 associated with the transition from /r/ to /a/ and, for some speakers, a corresponding increase in intensity. Likewise, segment durations were based on acoustic landmarks that could be clearly delineated. The offset of /d/ and onset of /r/ were assumed to be synchronous and correspond to the onset of high amplitude energy in F1. The CC-A interval was left-delimited by the mean of the midpoints of acoustic segments. Stability-based statistics, the RSD and SD of the three target intervals, were computed for each speaker. Of the 98 speakers, 83 showed CC-A stability; 13 showed RE-A stability; 2 had equal RSD for the RE-A and CC-A intervals.

**Fig 11:**
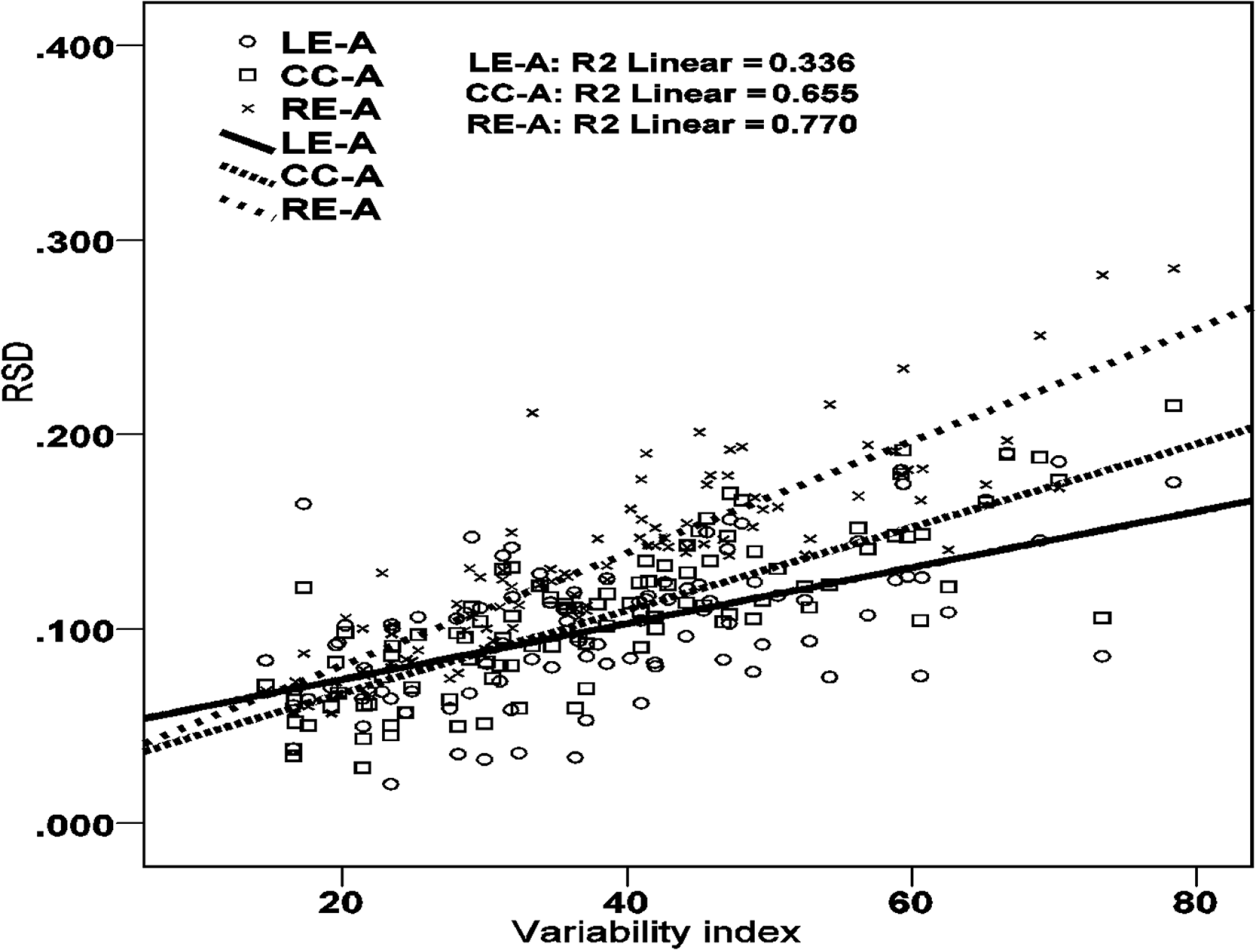
Interval stability dynamics for English acoustic data. Regression lines fit to RSDs of LE-A, CC-A, and RE-A intervals, y-axis, plotted against the standard deviation of the right edge to anchor interval for 96 speakers productions of the *raw~draw* dyad. The patterns in the regression lines correspond to the complex onset dynamic (Fig 7, right). Regression fits are significant at the *p* < .01 criterion for all intervals.

Fig 11 plots the RSD of target intervals against the variability index for the *raw~draw* dyad as produced by 98 talkers in the AusTalk corpus. Each set of three intervals comes from a different speaker. Regression lines were fit to the LE-A, CC-A, and RE-A intervals across speakers. As with the articulatory data above (Fig 10), the variability index was a significant predictor of RSD for each of the target intervals (LE-A [*B* = .580, *t*(97) = 6.971, *p* < .001]; CC-A [*B* = .809, *t*(97) = 13.499, *p* < .001]; RE-A [*B* = .878, *t*(97) = 17.944, *p* < .001]). The pattern in the regression lines corresponds to the prediction of the complex onset model (Fig 7 and right) and the articulatory data from the X-ray microbeam corpus of American English (Fig 10, right). At low levels of variability, the RSD of the CC-A is lower than the RE-A and LE-A intervals. As variability increases, we observe again a crossover point whereby the LE-A interval becomes more stable than the CC-A interval. The pattern found in the acoustic data reflects the unique dynamic characteristic of complex syllable onsets.

Although the precise mapping between articulatory and acoustic events remains a topic of on-going research, our preliminary results suggest that the modelling paradigm we have developed may be extended to acoustic data. This extension would put large and diverse populations within reach of our analytical tools enabling work on normal language development as well as the detection and evaluation of language impairment or developmental delay.

## Conclusion

Drawing on notions from complex systems, stochastic modelling, and generative phonology, we developed a formal paradigm linking qualitative phonological organization, expressed in terms of syllables, to continuous expressions of that organization in the movements of speech organs. Our models leveraged the use of stochastic noise to derive continuous predictions from discrete phonological variables. Embedded within our framework, syllables function to structure variability in phonetic measurements. Distinct syllabic organizations, as in parses of a segmental string into simplex vs. complex onsets, prescribe unique temporal dynamics. We used these dynamics to distinguish syllabic organization in Arabic and English, two languages argued to parse similar segmental strings into different syllabic organizations. Our models generated consistent predictions across a range of datasets collected at different labs and under different conditions, including cases in which phonetic heuristics for syllables are known to break down. Moreover, model predictions proved resilient to multiple sources of variability in the data including measurement variability, speaker variability, and contextual variability. The approach therefore provides rigorous new methods for evaluating syllabic organization. More broadly, it underscores the value of an emerging perspective on the relation between discrete and continuous aspects of a cognitive system—namely, that qualitatively different states of cognitive organization can be discerned in continuous data because they structure variability in different ways.

## Supporting Information

S1 File X-Ray Microbeam Data, subjects and tasks. (DOCX)

S2 File Permission to reproduce Fig 2. (TXT)
