# Supplementary material for: Stochastic Time Models of Syllable Structure
Source: PLoS One. 2015 May 21;10(5):e0124714. doi: 10.1371/journal.pone.0124714 (PMC4440707; doi:10.1371/journal.pone.0124714)
Supplement: S1 File — (DOCX) [file pone.0124714.s001.docx]

# Appendix A – X-Ray Microbeam Data, subjects and tasks

|  | row | grows | before | upon | about | between | across | hispanic |
| --- | --- | --- | --- | --- | --- | --- | --- | --- |
| Task(s) | 57 | 56 | 30, 32, 46, 48, 55, 64, 78 | 11, 12 | 7, 11, 18(x2), 41, 45, 76, 95 | 19, 28, 40, 74, 86, 101 | 19, 27, 28, 37, 46, 47, 68, 71, 80, 85, 90 | 7 |
| Total Subjects | 25 | 25 | 32 | 40 | 34 | 37 | 25 | 21 |
| Subject Ids | 42  55  60  34  33  44  56  36  30  59  31  21  13  40  61  54  27  52  35  57  48  43  37  45  39 | 13  42  56  41  35  31  28  61  57  30  39  26  34  55  20  59  48  63  58  40  12  44  53  43  54 | \| 12 36  13 37  14 40  15 42  18 45  19 46  20 49  21 51  24 52  28 55  29 56  31 59  32 60  33 61  34 62  35 63 \| \| --- \| | \| 11 36  12 37  13 39  14 40  15 41  16 42  18 43  20 44  21 48  24 49  25 51  26 52  27 53  28 54  29 55  30 56  31 58  33 60  34 62  35 63 \| \| --- \| | 11 40  12 41  15 44  16 46  18 48  20 49  21 51  25 52  27 53  28 54  29 55  31 56  33 57  34 59  35 60  36 61  37 63 | \| 11 37  12 39  13 41  14 42  15 43  16 44  18 45  19 46  24 48  25 49  27 51  29 52  30 53  32 54  33 57  34 58  35 59  36 62  63 \| \| --- \| | \| 11 31  12 33  13 36  18 39  20 41  21 44  24 45  25 54  26 56  27 58  29 60  30 62  63 \| \| --- \| | \| 15 30  16 32  18 34  19 41  21 44  25 48  26 49  27 51  28 52  29 54  63 \| \| --- \| |
